# Modulation of Drug Release in Anticancer Therapy: Recent Advances, Challenges, and Emerging Drug Delivery Platforms

**DOI:** 10.3390/pharmaceutics18060698

**Published:** 2026-06-05

**Authors:** Katarina Sokač Pogrmilović, Gordana Matijašić, Krunoslav Žižek

**Affiliations:** University of Zagreb, Faculty of Chemical Engineering and Technology, Trg Marka Marulića 19, 10000 Zagreb, Croatia

**Keywords:** tailored medicine, anticancer therapy, drug release, polymeric solid dispersions, cyclodextrin-based inclusion complexes, metal–organic frameworks, stimuli-responsive delivery, nanocarriers

## Abstract

Achieving precise control over anticancer drug release remains one of the key challenges in modern pharmaceutical development, as it directly determines therapeutic efficacy, systemic toxicity, and patient outcomes. This review critically evaluates recent advances in three major formulation strategies: polymeric solid dispersions, cyclodextrin-based inclusion complexes, and metal–organic frameworks (MOFs), with a particular focus on their capacity to tailor anticancer drug release. Over the past decade, polymeric solid dispersions and cyclodextrin-based carriers have played a central role in improving the dissolution and bioavailability of poorly water-soluble anticancer agents, while also enabling modified release profiles through rational formulation design. Increasing structural complexity, including ternary systems and supramolecular assemblies, reflects a shift toward more controllable delivery platforms. In recent years, MOFs have emerged as highly adaptable porous materials capable of supporting controlled and stimuli-responsive release. The integration of imaging agents, magnetic components, and photothermal functionalities has further enabled the design of multifunctional and theranostic platforms. Taken together, these technologies reflect a shift from conventional solubility enhancement toward structurally engineered systems designed to achieve predictable and controlled drug release. Continued advances in material design and formulation strategies are expected to further refine release kinetics and support the development of next-generation anticancer therapies aligned with the growing demand for precision medicine.

## 1. Introduction

In the 21st century, cancer represents a persistent global health challenge despite major therapeutic advances. It accounts for approximately one in six deaths globally and one in four deaths from noncommunicable chronic diseases [1]. Although cancer is predominantly observed in adults over the age of 50, the global incidence of early-onset cancer diagnosed before the age of 50 has been increasing. Moreover, the adverse effects of associated cancer treatments may result in additional health complications in later adulthood [2]. Cancer can take over one hundred distinct forms, as it results from the uncontrolled proliferation of different types of cells in the body. Carcinomas, malignancies of epithelial cells, account for about 90% of human cancers. Sarcomas, rare in humans, arise from connective and fibrous tissues. Leukemias and lymphomas, representing roughly 8% of malignancies, originate from blood-forming and immune system cells [3]. Advances in understanding carcinogenesis, cell biology, and the tumor microenvironment have translated into substantial therapeutic progress over the past 30 years. Nevertheless, many cancers remain fatal due to the limitations of current therapeutic strategies and unfavorable drug properties [4]. Thus, controlled drug release has emerged as a central design principle in modern anticancer therapy, as it directly governs therapeutic precision, systemic toxicity, and overall treatment outcomes. Achieving predictable release, however, remains challenging for many anticancer agents, particularly those with unfavorable physicochemical properties [5].

It is estimated that approximately 90% of drugs entering clinical development fail, primarily due to insufficient clinical efficacy (40–50%), toxicity (30%), poor pharmacokinetic and chemical properties (10–15%), and lack of commercial need or inadequate strategic planning (10%) [6]. A drug’s bioavailability, defined as the proportion of an administered dose that reaches systemic circulation, is a critical determinant of its therapeutic effectiveness, safety, and development potential [7]. Factors such as low solubility, limited gastrointestinal absorption, extensive first-pass metabolism, and efflux transporters can reduce bioavailability, contributing to the high failure rate of drug candidates during clinical development [8]. To predict and address these challenges, the Biopharmaceutical Classification System (BCS) categorizes active pharmaceutical ingredients into four classes (I–IV) based on their solubility and permeability [9]. Most new anticancer drug candidates fall into BCS Class II or IV. Class II compounds are characterized by high permeability but poor solubility, whereas Class IV compounds exhibit both low solubility and low permeability, posing significant challenges for oral bioavailability and successful clinical development [10]. For orally administered drugs, solubility is particularly important, since a drug must dissolve in gastrointestinal fluids before its absorption into the bloodstream. Poorly soluble drugs often require higher doses to reach therapeutic concentrations, which can increase the risk of toxicity and side effects [11]. These challenges are particularly pronounced for anticancer drugs, which often exhibit narrow therapeutic windows and require precise control over systemic exposure. Accordingly, the ability to modulate drug release has become fundamental to modern anticancer drug delivery. To overcome these problems, innovative formulation and delivery strategies are essential, with a focus on advanced carrier systems for anticancer drugs.

A wide range of carriers has been developed to enhance anticancer drug release and optimize release profiles. These include natural and synthetic polymers approved for human use [12,13], macrocyclic receptors with the ability to form supramolecular inclusion complexes with drug molecules [14], and metal–organic frameworks (MOFs), which are porous structures that can encapsulate drugs but are still in preclinical studies [15]. In polymeric drug delivery systems, polymers serve as carriers that encapsulate and transport drugs, while enabling enhanced and controlled release, biocompatibility, and adaptable chemical functionality [16]. Representative formulations include micelles [17], nanoparticles [18], solid dispersions [19], hydrogels [20], and nanofibers [21]. Macrocycle-based supramolecular drug delivery systems are formed through dynamic host–guest interactions between the hydrophobic cavities of the macrocyclic receptors and hydrophobic drug molecules [14]. Representative examples include crown ethers [22], cyclodextrins [23], cucurbit[n]urils [24], calix[n]arenes [25], and pillar[n]arenes [26]. Among the extensively investigated platforms for anticancer drug delivery are MOFs, which can encapsulate therapeutic agents and release them at the desired target site. These coordinative systems are composed of organic ligands and metal ions or clusters linked through coordination bonds [27], with zirconium(IV), iron(III), and zinc(II) being the most commonly used ions in drug delivery applications [28]. These systems enable precise modulation of the release profiles of anticancer drugs and alter their mechanisms of release in diverse ways [29]. Additionally, by adjusting the physicochemical properties of the carriers, such as polymer composition, macrocycle cavity structure, or MOF porosity and surface functionality, these modifications collectively enhance drug release rates, bioavailability, and tumor targeting [30]. However, a systematic and release-oriented comparison of these platforms remains insufficiently addressed in the current literature.

This review provides a critical overview of recent innovations in anticancer drug delivery systems, with a particular focus on polymeric solid dispersions, and cyclodextrin- and MOF-based formulations, published from 2015 to 2025. These three platforms were selected because they represent formulation strategies with different levels of structural complexity, ability to modulate drug release, and proximity to clinical application. This rationale enables direct comparison between established and emerging formulation strategies, allowing the review to evaluate how increasing carrier complexity may expand the possibilities for release control, while also introducing additional translational challenges. Polymeric solid dispersions are established formulation platforms in which polymer selection, drug–polymer interactions, and matrix structure affect both dissolution enhancement and release kinetics. In cyclodextrin-based formulations, host–guest complexation is used to improve solubility and modulate drug release, whereas MOFs offer highly tunable porous architectures capable of controlled and stimuli-responsive delivery. Comparing these systems therefore enables an evaluation of how carrier design affects drug liberation, from rapid dissolution enhancement to sustained, controlled, or stimuli-responsive release. For each category, we describe its development and summarize the most widely used formulation methods together with their impact on drug release profiles. Representative examples of their applications across various anticancer therapies are presented, highlighting how modulation of drug release may help maintain therapeutically relevant exposure, reduce premature or excessive drug release, and ultimately contribute to improved therapeutic efficacy and reduced adverse effects. Additionally, we discuss current limitations, translational challenges, and emerging directions for future research aimed at supporting the rational design of clinically relevant anticancer delivery platforms. In this review, anticancer therapy is considered primarily through the formulation and release behavior of anticancer agents. Ligand-mediated targeting and tumor microenvironment-responsive moieties are therefore addressed only where they directly contribute to carrier performance or site-selective drug release, rather than as independent topics of tumor-targeting nanomedicine.

## 2. Polymeric Solid Dispersions

**Figure 1:**
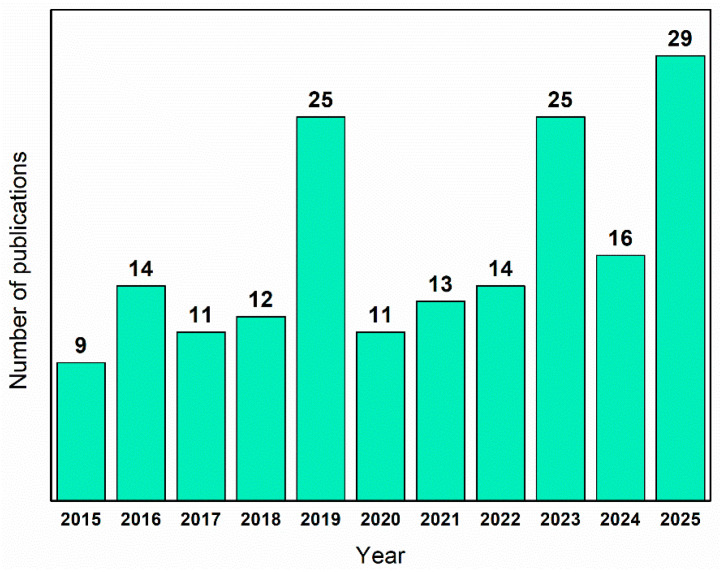
Number of publications indexed in the Scopus database between 2015 and 2025 related to the use of solid dispersions in anticancer drug formulation, obtained using the keywords “solid dispersion” and “anticancer drug.”

To evaluate the research activity related to the use of solid dispersions as carriers in antitumor therapy during the last decade (2015–2025), a literature search was conducted using the Scopus and PubMed databases. The keywords “solid dispersion” and “anticancer drug” yielded a total of 179 publications (Figure 1), comprising 154 research articles, 22 review papers, and 3 book chapters. The 154 research articles retrieved through this broader search were included in the screening process. The search was then refined by adding the term “polymeric” before “solid dispersion” to determine how many publications were explicitly indexed using this terminology, which resulted in 23 papers, including 17 research articles, 5 review papers, and 1 book chapter. Therefore, the narrower search was used only as a terminology-based refinement and not as an exclusion criterion. Although polymeric solid dispersions are a well-established strategy for improving drug solubility and bioavailability, they remain the least represented among the three formulation strategies discussed in this review over the last decade. In the anticancer drug studies from this period reviewed here, these systems were mainly investigated as solubility- and dissolution-enhancing platforms, while their release-modulating potential depended on polymer composition, matrix structure, and diffusion-, swelling-, or erosion-controlled mechanisms.

To the best of the authors’ knowledge, the first documented attempt to prepare a polymer-based solid dispersion of an anticancer drug dates back to 1993, when Du and Vasavada successfully developed solid dispersions of etoposide with polyethylene glycol (PEG) 8000 using the fusion method [31]. Etoposide, a topoisomerase II inhibitor commonly used in the treatment of lung cancer, induces DNA strand breaks that promote apoptosis in rapidly proliferating cancer cells [32]. In dissolution studies conducted in water at 37.0 ± 0.5 °C, the formulations demonstrated a pronounced enhancement in drug dissolution rate: solid dispersions with drug-to-polymer ratios of 1:20, 1:30, and 1:40 achieved complete etoposide release within one minute, whereas the pure drug required approximately 30 min for complete dissolution [31]. These findings were significant in demonstrating the potential of polymeric carriers to improve the dissolution of poorly soluble anticancer drugs. While early formulations primarily demonstrated rapid dissolution enhancement, subsequent research has progressively emphasized polymer matrices that enable controlled or sustained release. This shift reflects the need to moderate peak plasma concentrations and improve patient tolerability, particularly for potent anticancer agents. In the last decade, this issue has been addressed using biocompatible and functional polymers designed not only to enhance overall drug liberation but also to enable controlled and predictable release kinetics, ensuring steady systemic exposure, reduced side effects, and less frequent dosing. In addition to synthetic and natural polymers, various additives such as surfactants have also been incorporated to form ternary solid dispersions, further improving drug solubility and release performance. Apart from the polymer matrix and the drug, these ternary systems typically contain a third component, most commonly a surfactant or a small molecular excipient, which acts as a stabilizer or solubilizer. A summary of the anticancer agents, polymers, and preparation methods reported during this period is presented in Table 1.

A review of the studies summarized in Table 1 indicates that the most frequently reported formulations involve the development of amorphous solid dispersions, ternary systems combining the active pharmaceutical ingredient with one or more polymers and surfactants, or polymer blends designed to enhance the solubility, stability, and dissolution behavior of poorly water-soluble anticancer agents. Among the polymers, polyvinylpyrrolidone (PVP), hydroxypropyl methylcellulose (HPMC), PEG, Eudragit^®^ variants, Soluplus^®^, and poloxamers are the most widely used carriers, while surfactants such as Tween 80, sodium dodecyl sulfate (SDS), and D-α-tocopheryl polyethylene glycol succinate (TPGS) are usually incorporated to further improve wetting and drug dispersion. Over the past decade, there has also been a growing application of solid self-emulsifying drug delivery systems (S-SEDDS), which combine the advantages of lipid-based delivery and solid dispersion technology to enhance the physicochemical and biopharmaceutical properties of anticancer drugs. Curcumin remains one of the most extensively investigated anticancer compounds, serving as a model drug for optimizing formulation parameters. In addition to conventional preparation methods such as solvent evaporation, spray drying, and freeze drying, emerging techniques like electrospinning have been increasingly explored for their ability to produce nanofibrous systems with high surface area and controlled release characteristics. Furthermore, there is a rising interest in environmentally sustainable and solvent-free approaches, particularly mechanochemical activation, which offers a green alternative for producing amorphous solid dispersions without the use of organic solvents. The following subsections provide a more detailed overview of the most widely used formulation strategies, highlighting both traditional and novel approaches aimed at improving the solubility, dissolution rate, and bioavailability of anticancer agents.

### 2.1. Amorphous Solid Dispersions

**Figure 2:**
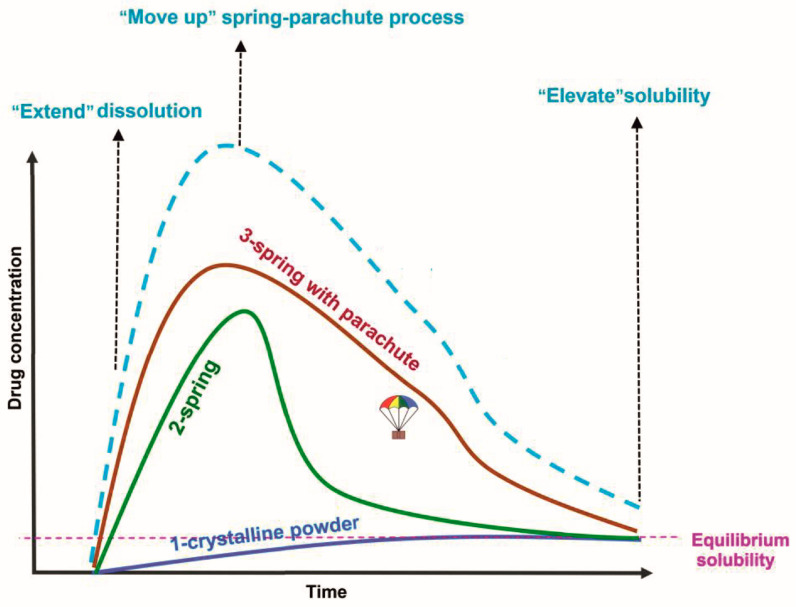
Schematic representation of the “spring and parachute” effect. Adapted from H. Yu et al. [58]; licensed under CC BY.

When a drug is present in the amorphous state, its free energy is higher compared with the corresponding crystalline form, rendering it thermodynamically unstable and prone to recrystallization. To mitigate this tendency, it is necessary to incorporate an excipient, most commonly a polymer, that reduces molecular mobility and thereby inhibits drug recrystallization [55]. Such formulations play a critical role in enhancing the solubility of poorly soluble drugs and in modulating their release profiles. However, the improved dissolution of the amorphous drug can generate a supersaturated solution, from which the drug may recrystallize and thus limit its absorption. The incorporation of a polymer helps suppress or delay precipitation by maintaining the drug in a molecularly dispersed state [56]. A commonly cited description of the improved dissolution behavior of amorphous solid dispersions is the “spring and parachute” effect (Figure 2). In this concept, the “spring” corresponds to the rapid initial dissolution that generates a supersaturated drug concentration, whereas the “parachute” refers to the ability of the system to maintain this supersaturation, which is essential for enhanced absorption and bioavailability. If the amorphous drug crystallizes too quickly once in solution, the supersaturation cannot be maintained, and the “spring and parachute” effect is essentially lost [57].

Polymeric amorphous solid dispersions were the most frequently applied strategy for enhancing the solubility and dissolution performance of anticancer drugs in this group during the reviewed period. Most of the investigated compounds predominantly belonged to BCS class II (andrographolide, apalutamide, apigenin, bicalutamide, dasatinib, erlotinib hydrochloride, flutamide, ibrutinib, lapatinib ditosylate, tacrolimus, telaprevir), characterized by high intestinal permeability but low aqueous solubility. Several anticancer agents fell within BCS class IV (curcumin, docetaxel, elacridar hydrochloride, kaempferol, methotrexate, naringin, paclitaxel), exhibiting both poor aqueous solubility and limited intestinal permeability. All investigated samples demonstrated significant improvements in the total amount of drug dissolved compared with the pristine drugs or their physical mixtures with polymers.

For andrographolide, the incorporation into an amorphous solid dispersion with Eudragit^®^ S100 was reported to enhance dissolution through amorphization, increase apparent solubility, and stabilize drug–polymer interactions. Drug dissolution improved substantially, increasing from roughly 40% for the untreated drug to nearly complete release from the amorphous solid dispersion [33]. The pH-responsive nature of Eudragit^®^ S100 facilitated rapid andrographolide release in simulated intestinal fluid at pH 6.8 and simulated colonic fluid at pH 7.4, with slightly higher final release observed at pH 7.4. This behavior is consistent with the pH-dependent hydration, swelling, and dissolution of Eudragit^®^ S100, which becomes more soluble under higher-pH intestinal and colonic conditions. As the polymer matrix hydrates and dissolves, drug liberation from the amorphous dispersion is promoted, while molecular dispersion and hydrogen bonding between andrographolide and the polymer reduce aggregation and improve wettability. These combined effects explain the markedly enhanced dissolution compared with the untreated drug [59]. Despite these enhancements, the release behavior appeared to be primarily diffusion-controlled, likely due to the viscosity of the hydrating polymer matrix [60]. For apalutamide, dissolution was examined under conditions with and without surfactant to compare drug release in different media. The amorphous form displayed markedly improved dissolution compared with the crystalline drug, while amorphous solid dispersions showed an additional enhancement, especially at lower drug loads. The release profiles varied depending on the presence of surfactant and on the polymer used, highlighting the role of polymer-mediated solubilization in achieving improved dissolution [34]. Co-milling of bicalutamide with either PEG 6000 or Poloxamer 407 led to up to 11-fold enhancement of dissolution. Improved dissolution was driven by two complementary mechanisms: increased wettability due to a decreased contact angle in PEG 6000 systems, and solubilization of the drug within nanoaggregates formed by the surface-active Poloxamer 407. Particle size reduction occurred but was not the primary factor, while structural modifications and reduced crystallinity further contributed to the enhanced release [36]. For curcumin, Eudragit^®^ EPO-based amorphous solid dispersions markedly improved dissolution compared with crystalline drug and physical mixtures (<4% in 2 h). Solid dispersions achieved rapid initial release (30–45% within 10–15 min), with drug-to-polymer 1:5 formulation showing the highest performance (90% after 10 min and complete dissolution after 2 h). The improvement was attributed to amorphization, reduced aggregation, and enhanced wetting [37]. Dasatinib solid dispersions also exhibited superior dissolution profiles. The most amorphous formulations released over 70% of the drug within 120 min, whereas the crystalline drug showed the lowest release. Semi-crystalline dispersions performed slightly worse, reflecting incomplete lattice disruption. Milling conditions influenced amorphization efficiency, with zirconium oxide jars yielding better dissolution outcomes [40]. Elacridar hydrochloride showed polymer-dependent improvements [43]. PVP K-30-based dispersions enhanced initial dissolution (up to 52%) but precipitated rapidly. PVP/VA 64 provided minimal enhancement (<10%), likely due to lower hydrophilicity [61]. Soluplus^®^ dispersions achieved moderate but sustained dissolution (up to 53%), attributed to micellar solubilization, and maintained better release than the crystalline drug over prolonged testing [62]. For erlotinib hydrochloride, no dissolution data were provided, but solid-state analyses confirmed complete amorphization in all polymer-based dispersions [44]. The reduced cohesive energy density and decreased Hildebrand solubility parameters indicated weaker intermolecular interactions and improved miscibility, suggesting an increased tendency for the drug to disperse and dissolve more readily compared with the crystalline form [63]. Flutamide showed very poor dissolution in its crystalline form (<9% in 5 h), with only minimal improvement in physical mixtures. Incorporation into amorphous solid dispersions markedly enhanced dissolution, particularly with PVP, where 69–95% of the drug dissolved within the first hour and high concentrations were maintained for 2 h. PEG also produced a strong initial enhancement (60–91% at 60 min), though followed by a decline in dissolved drug. HPMC and Eudragit^®^ EPO offered more modest improvements (35–55% over 5 h) [45]. The superior performance of PVP and PEG correlated with stronger drug–polymer interactions and complete amorphization, supporting their greater solubilizing capacity compared with the other polymers [64]. Ibrutinib showed a major improvement in solubility when formulated with different types of poloxamers. The optimized system achieved almost complete dissolution in acidic and mildly acidic media, reaching almost 100% of the dose in pH 1.2 and pH 4.5, a substantial enhancement over the crystalline drug and previously reported amorphous solid dispersions [46]. Poloxamer-containing dispersions were especially effective, providing rapid and extensive solubilization indicative of a strong potential to increase bioavailability [65]. Lapatinib ditosylate displayed strong pH-dependent solubility, dissolving moderately in 0.1 N HCl but showing extremely low solubility in water and almost no solubility in phosphate buffer at pH 6.8. Incorporation into PVP nanofibers led to a significant improvement, enabling almost complete dissolution in acidic medium and over 60% dissolution even at pH 6.8, in contrast to the crystalline drug [50]. The enhanced solubility was attributed to the amorphous state achieved during electrospinning and the high surface area of the nanofibrous matrix [66]. This system represents an excellent example in which amorphization not only enhances solubility but also alters the drug release profile: dissolution in phosphate buffer occurs as an immediate-release process, whereas in acidic medium the release is controlled and prolonged, demonstrating pH-responsive behavior [50]. Binary solid dispersions of naringin with Kollidon^®^ VA 64 or Soluplus^®^ markedly improved drug solubility, achieving 73–95% dissolution within 120 min and outperforming dispersions with Kollidon^®^ VA 30 or Poloxamer 188 [52]. Telaprevir showed very limited dissolution in its crystalline form, releasing only about 3% of the drug after 240 min. Milling increased the dissolution to around 9% due to reduced particle size and partial amorphization, although recrystallization diminished the overall benefit. Incorporation into hydrophilic polymers resulted in a markedly improved dissolution performance. Dispersion with PVP reached roughly 17% after 240 min, dispersion with PEG achieved a dissolution plateau of about 24% within 120 min, while dispersion with HPMC demonstrated the most pronounced enhancement, releasing 41% of the drug within 10 min and reaching 63% after 30 min [54].

**Figure 3:**
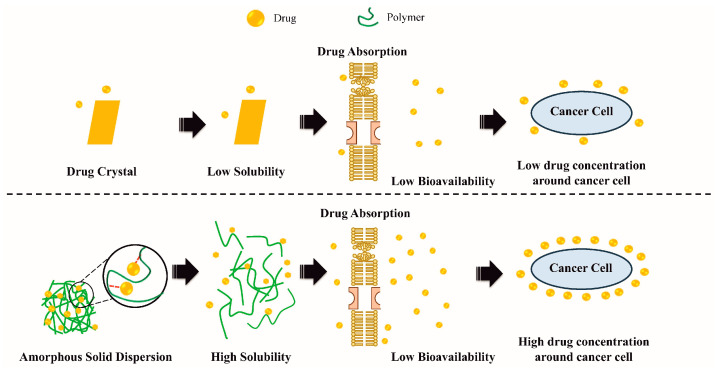
Proposed mechanism of bioavailability improvement in amorphous anticancer drugs formulated as amorphous solid dispersions. The amorphous form exhibits higher solubility and absorption compared with the crystalline form. Reproduced from A. Budiman et al. [55]; licensed under CC BY.

Collectively, these examples highlight the central role of polymer matrices in modulating drug behavior within solid dispersions. Through weak intermolecular interactions such as hydrogen bonding, van der Waals forces, hydrophobic associations, or electrostatic interactions, polymers reduce the molecular mobility of the embedded drug, thereby stabilizing the system and, in certain formulations, enabling modified or partially controlled release [67]. Depending on the physicochemical nature of both the drug and the polymer, release may proceed through diffusion from non-biodegradable matrices, swelling-controlled transport from hydrophilic polymers or erosion-controlled mechanisms in degradable systems [68]. By tailoring these processes, polymeric amorphous solid dispersions not only enhance solubility and dissolution rates but also allow tunable adjustment of the release profile, which can mitigate fluctuations in plasma concentration, reduce dosing frequency, and ultimately lower the incidence of adverse effects (Figure 3).

### 2.2. Ternary Solid Dispersions

**Figure 4:**
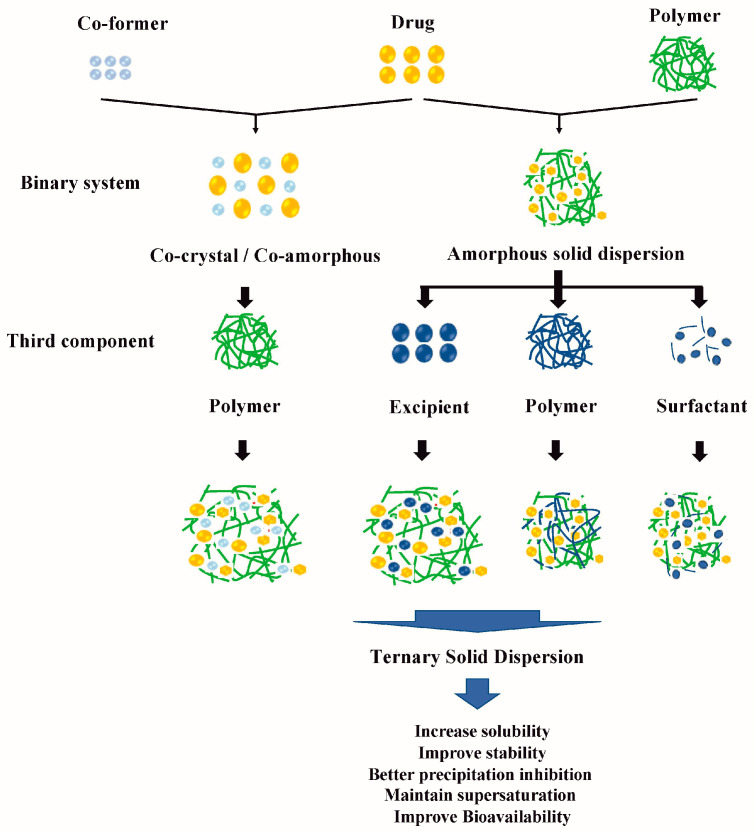
Possible components of ternary solid dispersions. Reproduced from A. Budiman et al. [73]; licensed under CC BY.

Ternary solid dispersions consist of an active pharmaceutical ingredient dispersed within two distinct carrier components in the solid state, forming a multicomponent delivery matrix. The additional component, commonly a surfactant, a second polymer, or another functional excipient, is introduced to further optimize the formulation [69]. This approach aims to achieve greater improvements in solubility, physicochemical stability, bioavailability, and more predictable drug-release performance compared with conventional binary dispersions (Figure 4). The most common ternary approach involves the incorporation of either a second polymer or a surfactant. Additional polymers, which are often selected from those already described, are combined in various ratios to leverage differences in their physicochemical properties and thereby broaden the functional performance of the system. Their combined action can improve and prolong supersaturation, inhibit nucleation and crystal growth, and increase formulation stability. Polymers contribute to these effects by elevating the glass transition temperature and reducing the molecular mobility of the drug, collectively preventing recrystallization and enabling more consistent drug release [70]. In the case of surfactants, their amphiphilic nature markedly improves the drug’s compatibility with the polymeric matrix. Their capacity to form micelles further increases drug wettability and apparent solubility, thereby affecting both dissolution rate and overall release behavior [71]. Due to their favorable safety profiles, non-ionic surfactants such as Tweens and poloxamers are most commonly used [72]. In many formulations, surfactants act synergistically with polymers or adsorb onto the drug surface, reducing interfacial tension and enhancing its dispersion. The underlying mechanism primarily involves the adsorption of surfactant molecules onto polymer chains, resulting in improved stabilization of the drug within the solid dispersion system [69].

In research conducted over the last decade, several drugs have been formulated with additional polymers or surfactants to improve release profiles, including curcumin, docetaxel, and lapatinib ditosylate. Moreover, some drugs previously investigated as binary solid dispersions, such as elacridar hydrochloride, erlotinib hydrochloride, and naringin, were also studied as ternary dispersions with the addition of another polymer or surfactant (Table 1) to compare solubility and dissolution outcomes. Most of these drugs belong to BCS class IV, with low aqueous solubility and limited intestinal permeability, except for lapatinib ditosylate and erlotinib hydrochloride. The tendency to use ternary solid dispersions reflects the recognized advantages of this approach over binary systems, particularly for BCS class IV compounds, where dissolution-limited absorption remains a primary barrier to therapeutic performance.

Ternary solid dispersions of curcumin with PVP K-30 and Tween 80 were incorporated into floating tablets. These formulations markedly enhanced solubility and dissolution, achieving approximately 241-fold higher release compared with tablets containing pristine curcumin. The optimal composition provided both improved solubility and stability, demonstrating the benefit of combining polymers and surfactants in ternary systems [38]. Docetaxel solid dispersions incorporating hydrophilic polymers and the surfactant SDS demonstrated markedly enhanced dissolution compared with the pure drug. Among the tested formulations, the dispersion prepared with Eudragit^®^ L100 (drug:polymer:surfactant = 1:3:0.01) showed the highest release, achieving around 28% at the first time point and 56% after 300 min, representing a more than fivefold improvement over crystalline docetaxel. Increasing the proportion of Eudragit^®^ L100 further enhanced drug dissolution [42]. Lapatinib ditosylate showed limited dissolution, reaching approximately 32% after 45 min. Nanosizing and dispersion within hydrophilic stabilizers markedly improved release in all prepared formulations. The formulation containing PVP K-30 and SDS provided the highest enhancement, with drug release more than doubled at both sampling points and exceeding even the marketed Tykerb^®^ product. Drug formulations stabilized with HPMC E5 or L-HPC also showed substantially increased dissolution, though to a lesser extent. A physical mixture of lapatinib, PVP K-30, and SDS produced only minimal improvement, which indicates that the enhanced dissolution arises primarily from the nanoscale dispersion of lapatinib within the polymer matrix, rather than from simple mixing of the components [49]. Collectively, these findings highlight the critical role of surfactant-mediated stabilization in maximizing the performance of ternary dispersions.

The comparison of binary and ternary dispersions containing previously described drugs elacridar hydrochloride, erlotinib hydrochloride, and naringin showed significant improvements with the addition of a surfactant. Namely, the inclusion of the surfactant consistently enhanced solubility and accelerated dissolution by improving wettability, promoting molecular-level dispersion within the polymer matrix, and stabilizing supersaturation compared with the corresponding binary systems. Ternary solid dispersions of elacridar hydrochloride demonstrated a clear advantage with the addition of SDS, which markedly improved both the degree and duration of supersaturation, leading to higher dissolution and significantly slower precipitation. In PVP K-30-based ternary dispersions, the addition of SDS produced dissolution levels far superior to those of the corresponding binary systems. The optimized formulation (drug:PVP K-30:SDS = 1:6:1) achieved over 90% dissolution, representing roughly a 90-fold increase compared with crystalline elacridar hydrochloride. Soluplus^®^-SDS ternary dispersions also showed substantial enhancement, attributed to more efficient micelle formation and reduced agglomeration [43]. Characterization of erlotinib hydrochloride solid dispersions revealed weak hydrogen bonding in the binary systems with PVP K-30 and PEG 4000, while interactions in the ternary system were negligible. Solubility parameter calculations using Hildebrand and Hansen models showed a progressive decrease in cohesive energy density from the pristine drug to the binary dispersions with PEG 4000 and PVP K-30, and further to the ternary system. The lowest Hildebrand parameter in the ternary system suggests reduced intermolecular forces, promoting better dispersibility and solubility [44]. The ternary solid dispersion of naringin combining Kollidon^®^ VA 64 and Soluplus^®^ further enhanced dissolution over the corresponding binary systems, achieving approximately 99% release within 60 min. Solid-state characterization confirmed conversion of the drug to the amorphous form. These findings highlight the effectiveness of polymer blends in improving solubility and dissolution, which was also reflected in enhanced in vitro antioxidant, antimicrobial, and cytotoxic activities compared with the pristine drug [52].

Ternary solid dispersions consistently demonstrated superior performance compared with their binary counterparts while enabling greater modulation of release behavior. The addition of a second polymer or surfactant enhanced solubility, maintained supersaturation, and reduced precipitation, leading to faster and more complete drug release. These systems leverage complementary polymer properties and surfactant-mediated solubilization to stabilize the amorphous state, improve wettability, and control release, ultimately offering a powerful strategy to overcome solubility-limited bioavailability of poorly water-soluble drugs. Careful selection of the third component and its ratio is critical, as these parameters strongly affect dissolution performance and stability. At the same time, the increased complexity of ternary systems can pose challenges in characterization and optimization, requiring thorough physicochemical and solid-state analyses to ensure consistent performance [73].

### 2.3. Solid Self-Emulsifying Drug Delivery Systems

**Figure 5:**
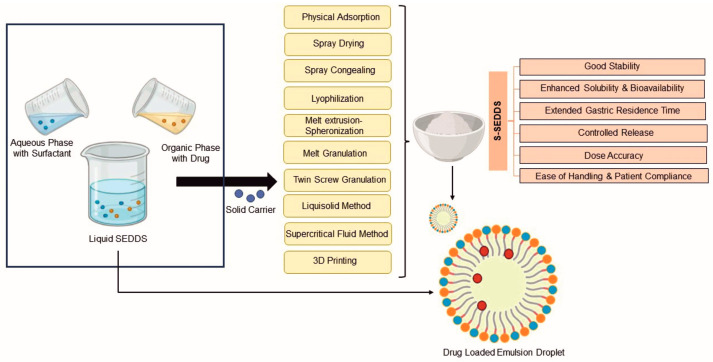
Basic principles and preparation methods of S-SEDDS. Reproduced from I. Govindan et al. [76]; licensed under CC BY.

S-SEDDS are included in this review because of their conceptual relationship with solid dispersions and their growing relevance in anticancer drug formulations over the past decade. Liquid SEDDS (L-SEDDS) suffer from several drawbacks during manufacturing and storage, such as limited stability, handling difficulties, and restricted formulation flexibility, which can ultimately reduce patient compliance. These limitations have driven considerable interest in S-SEDDS, which offer improved stability, reproducibility, patient acceptability, and easier process control [74]. S-SEDDS are generated by incorporating L-SEDDS into a suitable solid carrier and can be further processed into powders, pellets, capsules, or tablets. The choice of the solid carrier is a critical step: it must provide high liquid-loading capacity to efficiently absorb and retain the L-SEDDS, exhibit good flowability, and possess adequate mechanical strength for processing. Carrier hydrophilicity and porosity are especially important parameters for achieving controlled drug release [75]. Figure 5 summarizes the most commonly used solidification methods for S-SEDDS and highlights their advantages over L-SEDDS.

The role of the solid carrier in S-SEDDS extends far beyond simple solidification. Its physicochemical characteristics must be carefully tailored in accordance with the drug’s properties and the intended therapeutic performance. Incorporating L-SEDDS into a solid matrix also mitigates key drawbacks of liquid systems, such as gastrointestinal irritation caused by high surfactant concentrations and co-solvent migration into gelatin capsule shells, which can trigger precipitation of lipophilic drugs [77]. Common carrier classes include silica- and silicate-based materials, polymer-based carriers, mesoporous carbon, porous carbonate salts, clays, and carbohydrate-based matrices. Among these, polymeric carriers such as HPMC, sodium carboxymethylcellulose (Na-CMC), poloxamers, and cyclodextrins are frequently used due to their ability to effectively adsorb or immobilize L-SEDDS. Their tunable hydrophilicity, porosity, and mechanical properties enable precise modulation of drug release and overall formulation performance [75].

Two representative studies are summarized in Table 1. They involve methotrexate (MTX) and paclitaxel, both belonging to BCS class IV, and tacrolimus, a BCS class II compound. Transmission electron microscopy (TEM) of MTX nanodispersions confirmed the presence of spherical particles with a solid core, while the presence of TPGS micelles contributed to improved solubility and stabilization of the drug. Within this structured PVP K-30/TPGS matrix, MTX exhibited a markedly improved and more controlled release profile, characterized by diffusion-governed kinetics. Whereas the pristine drug showed an immediate burst release upon contact with dissolution medium, nanodispersions released MTX gradually and diffusively, following Higuchi kinetics consistent with a predominantly Fickian diffusion-controlled mechanism. Encapsulation in the polymer-surfactant network significantly slowed drug transport across the membrane and eliminated the initial burst effect, enabling sustained release over 48 h. The authors reported that the controlled release behavior resulted from the strong association of the hydrophobic drug within the nanodispersion core and the stabilizing, solubilizing effect of TPGS micelles, both of which act to restrict rapid drug diffusion and prolong its release [51]. Polyethylene glycol-poly(ε-caprolactone) (MPEG-PCL) micelles provided sustained release of both paclitaxel and tacrolimus compared with the rapid release observed from the native drugs. In phosphate-buffered saline with Tween 80, free paclitaxel released more than 70% within 48 h, whereas paclitaxel-loaded micelles and dual-loaded micelles released only about half that amount over the same period. A similar trend was observed for tacrolimus, confirming that micellar encapsulation markedly slows drug diffusion. Although release was slightly faster in serum-containing medium, micelles still maintained significantly prolonged release compared with free drugs. Importantly, co-loading paclitaxel and tacrolimus did not alter the release behavior of either agent, demonstrating that the micellar core can independently accommodate and sustain both drugs without competitive effects [53].

Overall, these findings highlight the therapeutic potential of S-SEDDS for anticancer drug delivery. By embedding poorly soluble drugs within structured polymeric or micellar matrices, S-SEDDS can markedly enhance solubility, minimize burst release, and achieve sustained, diffusion-controlled drug release profiles [78,79]. Their ability to stabilize hydrophobic molecules, reduce drug precipitation, and modify release kinetics makes them particularly valuable for compounds with unfavorable BCS classifications [80]. Moreover, S-SEDDS offer improved physical stability, better patient acceptability, and greater versatility compared with their liquid counterparts, all of which position them as promising platforms for next-generation anticancer therapeutics [81].

## 3. Cyclodextrin-Based Inclusion Complexes

**Figure 6:**
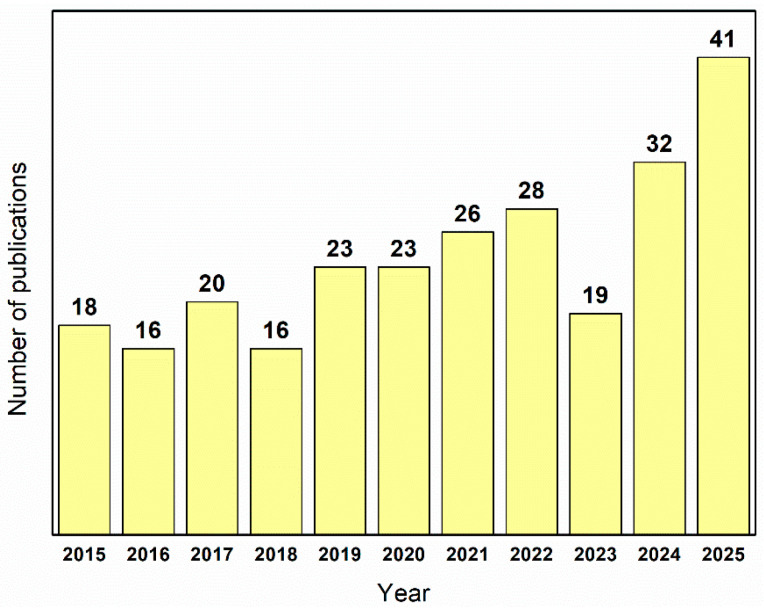
Number of publications indexed in the Scopus database between 2015 and 2025 related to the use of cyclodextrins as carriers in anticancer drug formulation, obtained using the keywords “cyclodextrin,” “inclusion complex,” and “anticancer drug.”

During the comprehensive literature search on cyclodextrin-based inclusion complexes of anticancer drugs, the query was specifically restricted to the terms “cyclodextrin,” “inclusion complex,” and “anticancer drug.” This search yielded 262 results (Figure 6), slightly more than the number obtained for solid dispersions. Among these entries, 233 were original research articles, 27 were review papers, one was a book chapter, and one publication had been retracted. The largest number of publications, 41 articles, appeared in 2025, indicating a continued and steadily increasing scientific interest in exploring inclusion complexes of natural cyclodextrins and their derivatives. This upward trend underscores the relevance of cyclodextrin-based systems for enhancing solubility, stability, and release characteristics of anticancer therapeutics, particularly for highly hydrophobic drug molecules. This growing attention is driven by the versatility of cyclodextrin chemistry, expanding possibilities for tailor-made derivatives and multifunctional delivery systems.

The earliest identified work on cyclodextrin inclusion complexes with anticancer agents dates back to 1990, when O. Bekers et al. reported their findings at the Second International Symposium on Pharmaceutical and Biomedical Analysis in York, UK [82]. In this study, the authors demonstrated that the anthracycline anticancer drugs doxorubicin (DOX) and daunorubicin are able to form inclusion complexes with γ-cyclodextrin (γ-CD). As previously discussed, the cavities of the other natural cyclodextrins (α- and β-CD) are too narrow to accommodate these bulky molecules, making γ-CD the only native cyclodextrin capable of encapsulating them [82]. However, no data were provided regarding whether incorporation into γ-CD altered the solubility of these drugs. A year later, J. J. Torres-Labandeira et al. investigated the solubilization of the anticancer agent pancratistatin using a wide panel of cyclodextrin derivatives [83]. Although direct dissolution in aqueous cyclodextrin solutions remained modest (0.1–1.2 mg mL^−1^), complexation with hydroxypropyl-β-cyclodextrin (HP-β-CD) markedly improved apparent solubility and produced the most stable complexes. By dissolving pancratistatin in a 50-fold excess of HP-β-CD in the presence of ammonia, followed by freeze drying, the authors obtained amorphous complexes that rapidly generated clear, supersaturated solutions upon reconstitution, reaching concentrations up to 9 mg mL^−1^. This represented a significant improvement compared with the drug’s intrinsic solubility (approximately 50 μg mL^−1^). Supersaturated solutions derived from HP-β-CD remained sufficiently stable for practical use in parenteral formulations, underscoring the potential of cyclodextrin complexation to dramatically increase the aqueous solubility of poorly soluble anticancer compounds [83]. Recent research has increasingly focused on designing cyclodextrin-based complexes that enable controlled release of anticancer agents [84]. Host–guest architectures can improve the loading efficiency of hydrophobic drugs and modulate their release by altering physicochemical properties, enhancing stability, and preventing premature degradation in biological environments [85]. Drug release from cyclodextrin cavities primarily follows a time-dependent dissociation mechanism, driven by dilution or competitive displacement, which is largely governed by the binding affinity between the drug and the cyclodextrin [86]. Understanding and tuning this balance between association and dissociation is therefore essential for developing effective delivery systems for anticancer therapy.

Representative examples of successful inclusion of anticancer drugs into various natural cyclodextrins and their derivatives over the past decade are summarized in Table 2. The emphasis was placed on studies demonstrating modified and controlled release profiles compared with the pristine drug, thereby highlighting the potential of these carriers as effective modulators of release behavior. Increasing attention is also being directed toward designing complexes capable of providing sustained, stimuli-responsive, or site-specific delivery, reflecting the broader shift toward smarter and more adaptable delivery platforms for anticancer therapy. During the screening process, studies based solely on computational methods, as well as those that did not provide experimental release profiles, were subsequently excluded from the final selection.

A review of the studies summarized in Table 2 shows that cyclodextrin-based inclusion complexes represent a versatile platform for improving the physicochemical and biopharmaceutical properties of anticancer drugs. The majority of formulations rely on β-CD and its hydrophilic derivatives, particularly HP-β-CD, methylated β-cyclodextrins (M-β-CD, DM-β-CD), sulfobutylether-β-cyclodextrin (SBE-β-CD), and an expanding range of functionalized or dendrimer-modified β-CD structures owing to their favorable safety profiles, aqueous solubility, and strong complexation capacity. In addition, γ-CD and specialized β-CD derivatives are increasingly employed for bulkier or highly hydrophobic molecules, emphasizing the importance of matching cavity dimensions and host–guest interactions to the structural characteristics of a given drug [131]. Among the studies summarized in Table 2, freeze drying was the most frequently reported method for obtaining solid inclusion complexes, particularly for heat-sensitive molecules, as it enables the formation of amorphous solids with superior solubility and rapid dissolution [132]. Other techniques such as kneading, solvent evaporation, and co-precipitation were also widely applied, each offering distinct advantages related to process simplicity, scalability, or environmental sustainability. Notably, both the choice of cyclodextrin derivative and the preparation method strongly affect drug release behavior, highlighting the central role of host–guest interactions in determining complex stability, binding affinity, and release kinetics [133].

In recent years, research has shifted beyond traditional binary inclusion complexes toward the development of delivery systems designed specifically to provide controlled, sustained, or stimuli-responsive release. Ternary complexes incorporating polymers (e.g., PVP K-30, HPMC), excipients, or bioactive additives have demonstrated improved stability, enhanced protection against degradation, and better release profiles [104,108,109,114,117]. Moreover, an increasing number of researchers are exploring advanced cyclodextrin-based nanostructures including nanosponges [134], nanohydrogels [135], and supramolecular polymeric networks [136], which offer improved drug-loading capacity and prolonged or environment-triggered release. Cyclodextrin-based nanosponges, particularly those produced through crosslinking of β-CD with carbonyl or carbonate linkers, have gained considerable attention due to their highly porous, three-dimensional architecture, which enables simultaneous encapsulation of hydrophobic and moderately hydrophilic molecules [137]. These systems often exhibit superior stability, sustained release behavior, and the ability to modulate pharmacokinetics even more effectively than simple inclusion complexes. Similarly, cyclodextrin-based nanohydrogels provide a hydrated, biocompatible matrix capable of maintaining drug supersaturation and enabling responsive release in reaction to pH, temperature, or enzymatic triggers [138]. Such emerging nanoarchitectures are of particular relevance for anticancer therapy, where controlled and site-specific delivery can substantially impact therapeutic efficacy and safety. These findings reflect a clear evolution of cyclodextrin-based systems from traditional solubilization tools toward multifunctional, structurally complex nanosystems capable of modifying release profiles, enhancing stability, and targeting specific biological environments [139]. To place the results obtained during the last decade in a broader scientific context, the following sections provide a systematic overview of natural cyclodextrins, their derivative forms, and the mechanistic principles governing inclusion complex formation, which are essential for interpreting the reported drug release phenomena.

### 3.1. Natural Cyclodextrin-Based Inclusion Complexes

**Figure 7:**
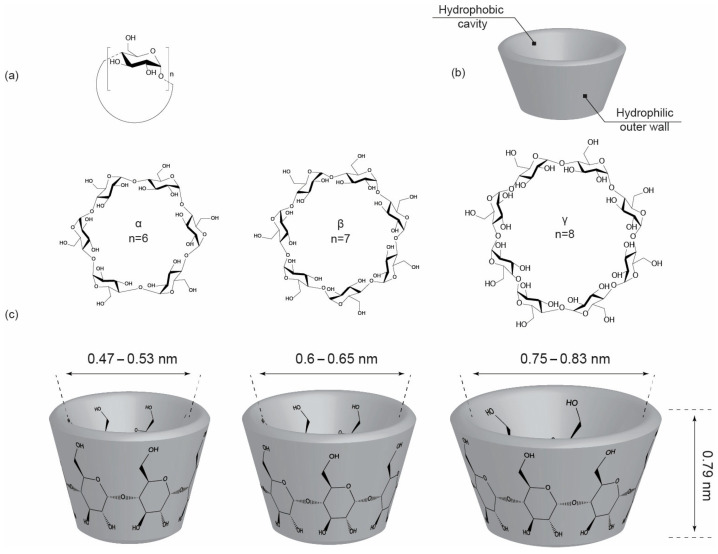
Natural cyclodextrins with corresponding hydrophobic cavity dimensions: (**a**) general chemical structure; (**b**) three-dimensional structure; (**c**) chemical structures and cavity dimensions of α-, β-, and γ-cyclodextrin, where n = 6, 7, and 8, respectively. Adapted from I. Spiridon et al. [142]; licensed under CC BY.

Cyclodextrins are cyclic oligosaccharides composed of varying numbers of glucopyranose units and capable of partially or fully embedding hydrophobic drug molecules within their hydrophobic cavity. They are produced by enzymatic degradation of starch using cyclodextrin glucosyltransferase. Natural cyclodextrins commonly used in pharmaceutical and food industries are α-, β-, and γ-CD, containing six, seven, and eight glucopyranose units, respectively (Figure 7). The U.S. Food and Drug Administration (FDA) has confirmed their safety. Therefore, they hold GRAS status (Generally Recognized as Safe) for oral dosage forms [140]. The outer surface of the cyclodextrin molecule is hydrophilic, which accounts for its relatively good water solubility, while the lipophilic inner cavity enables the formation of inclusion complexes with hydrophobic molecules. The established supramolecular interactions can markedly improve the solubility and stability of poorly soluble drugs, protect them from degradation, and modify their release profile. For detailed characterization of these inclusion complexes and for understanding the interactions between cyclodextrins and drug molecules, it is essential to use appropriate analytical methods [141].

The most commonly used natural cyclodextrin in pharmaceutical formulations is β-CD, primarily because its cavity size is more suitable for accommodating a wide range of drug molecules than the smaller α-CD. In addition, its relatively low aqueous solubility among natural cyclodextrins makes β-CD attractive for sustained release designs, particularly for hydrophobic anticancer agents [102,143]. In contrast, the large-scale use of γ-CD is limited by the high cost of the required enzymatic production and purification processes, which limits its broader application in pharmaceutical formulations [144]. Studies involving 5-fluorouracil (5-FU) and β-CD complexes incorporated into hydrogel matrices demonstrated clearly prolonged and controlled release, markedly slower than the free drug and further extended by increasing the guar gum content [87]. In liposomal systems, the HP-β-CD complex released 5-FU much faster than the corresponding β-CD complex (within 1 h at 41 °C), suggesting that β-CD provides slower and more sustained release compared with its more hydrophilic derivative [88]. Other studies have also compared drug release from inclusion complexes with natural cyclodextrins and their derivatives. For amlodipine and amygdalin, complexation with β-CD showed an initial burst release followed by a slower, diffusion-controlled phase with no significant differences compared with inclusion complexes of these drugs with β-CD derivatives [90,91]. For camptothecin, β-CD inclusion complexes enabled rapid and nearly complete drug release under physiological conditions, achieving almost full dissolution within 5 h, whereas the free drug released only about 20% over the same period [94]. Across natural cyclodextrins, release profiles range from rapid dissolution enhancement to sustained, stimulus-responsive delivery, depending primarily on formulation architecture. Among anticancer agents, curcumin has been investigated in a wide range of cyclodextrin-based delivery systems, resulting in distinctly different release profiles depending on formulation complexity. In simple β-CD inclusion complexes, curcumin release was primarily governed by improved aqueous solubility and amorphization, resulting in substantially faster and nearly complete dissolution within 6 h, whereas the free drug reached only about 50% release over the same time period under gastrointestinal pH conditions [96]. More advanced formulations, such as β- or γ-CD inclusion complexes incorporated into chitosan nanoparticles, exhibited pH-dependent and sustained release, achieving nearly complete curcumin release within about 90 min under acidic conditions that mimic tumor or intracellular environments [100]. In contrast, enzyme-responsive β-CD-curcumin systems enabled triggered drug release, where curcumin was released slowly in the absence of the enzyme but rapidly and completely upon enzymatic degradation of the cyclodextrin cavity [101]. Together, these studies demonstrate that curcumin release from cyclodextrin-based systems can be finely tuned from enhanced passive dissolution to pH- or enzyme-controlled delivery by increasing formulation complexity. β-CD inclusion complexes of diosmetin exhibited pH-dependent release from hydrogel matrices, with increasing release at higher pH values representative of intestinal conditions. Compared with co-grinding and kneading, microwave-assisted complexation provided the most controlled and sustained release [103]. Similarly, among the preparation methods of etoposide-β-CD inclusion complexes, solvent evaporation provided the highest release, followed by kneading, indicating more efficient complexation, reduced crystallinity, and improved wettability [113]. Similar to 5-FU, dasatinib showed significantly slower release from β-CD than from HP-β-CD, while increasing the amount of either cyclodextrin led to a higher total drug release [102]. DOX release from β-CD inclusion complexes showed sustained, pH-responsive behavior. In β-CD nanoparticles with quercetin, total DOX release reached approximately 74% at pH 7.4 and 91% at pH 4 over 96 h [107]. Similarly, in β-CD-containing polyamide nanofibers, release was slower initially (33–38% in 10 h) and reached 90–93% over four days, demonstrating that β-CD effectively controlled its release while allowing faster release under acidic, tumor-like conditions [108]. Spray-dried MTX inclusion complexes with HP-β-CD, β-CD, M-β-CD, and DM-β-CD showed substantially enhanced solubility and in vitro release compared with the free drug. Among them, DM-β-CD provided the highest release, with nearly complete drug liberation within 1 h, reflecting stronger host–guest interactions, amorphization, and reduced particle size, all contributing to faster and more efficient dissolution [118]. In contrast to MTX, all pinostilbene-loaded inclusion complexes exhibited markedly faster release than the free drug, with release strongly influenced by pH. After 24 h, the complexes achieved higher cumulative release, particularly at physiological pH, likely due to the amorphous state of the drug within the cyclodextrin cavity enhancing solubility and diffusion [123]. Raloxifene exhibited a notably slow and low release profile, with less than 10% dissolved within 120 min under simulated gastric conditions. Inclusion complexes with cyclodextrins effectively enhanced drug release, while co-precipitates with tartaric acid or azelaic acid further improved total dissolution. Co-precipitation proved more effective than milling or freeze drying in promoting drug release, consistent with solubility data [125]. Resibufogenin exhibited very slow dissolution, with only 18% and 31% released at 60 and 240 min, respectively. Physical mixtures with β-CD or HP-β-CD improved release slightly (50–53% at 240 min), whereas inclusion complexes (regardless of whether β-CD or HP-β-CD was used) achieved nearly complete dissolution within 5 min, owing to amorphization, enhanced wettability, and surfactant-like effects of the cyclodextrins [126].

A further advancement over classical complexes is represented by ternary systems, in which a third component is incorporated alongside the drug and cyclodextrin. Ternary inclusion complexes of docetaxel, exemestane, and lapatinib ditosylate with β-CD demonstrated markedly improved drug release compared with the free drugs and their binary counterparts. In all cases, physical mixtures showed only modest enhancement due to the wettability effect of β-CD, whereas complexation via kneading or freeze drying significantly increased the dissolution rates. The addition of a hydrophilic polymer, HPMC E5 for docetaxel and exemestane and PVP K-30 for lapatinib ditosylate, further promoted drug release by enhancing inclusion within the β-CD cavity, improving solubility and reducing crystallinity. For docetaxel, ternary complexes achieved 22% release at early time points and up to 45% after 48 h, while exemestane ternary complexes reached 74% release within 45 min and lapatinib ditosylate ternary complexes reached 65% release in the same period. These results indicate that ternary β-CD complexes with hydrophilic polymers can effectively modulate drug dissolution, providing faster and more complete release than binary complexes or physical mixtures [104,114,117].

In this field, the greatest number of studies has focused on the anticancer agent curcumin, followed by DOX. Unlike the dispersion techniques described in the previous section, this formulation approach is applicable to drugs from all BCS classes, although it is most commonly leveraged for poorly water-soluble compounds. Cyclodextrins therefore serve not only to enhance the solubility of poorly water-soluble class II and IV drugs, but their ability to provide prolonged and controlled release also broadens their application to a wider range of therapeutics. β-CD, the most frequently used natural cyclodextrin, has proven effective in both solubility enhancement and release control, while cyclodextrin derivatives are generally employed to further improve solubility and release rate, thereby significantly affecting the absorption and oral bioavailability of anticancer drugs.

### 3.2. Cyclodextrin Derivative-Based Inclusion Complexes

**Figure 8:**
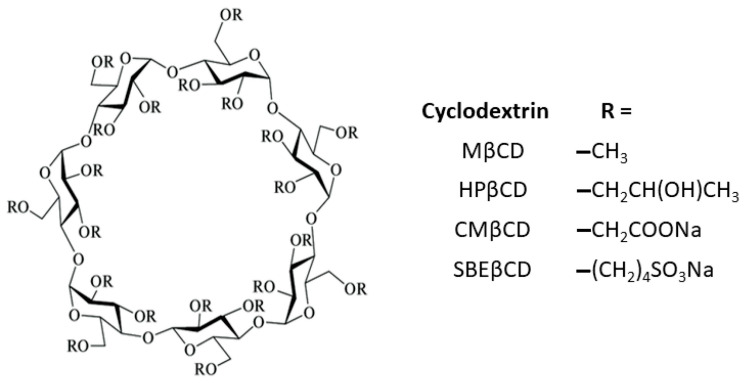
Overview of the most common cyclodextrin derivatives. Adapted from V. Aiassa et al. [146]; licensed under CC BY.

Cyclodextrins are typically modified at the three hydroxyl groups present on each anhydroglucose unit. Among them, the hydroxyl group at the C6 position exhibits the highest basicity and is the most accessible for chemical modification, whereas the C2 hydroxyl is the most acidic, and the C3 hydroxyl is the most sterically hindered. These differences in reactivity enable selective functionalization to obtain desired cyclodextrin derivatives. Today, various functionalized cyclodextrins are available (Figure 8), including nonionic hydrophilic, mildly lipophilic, and ionic forms. The significance of these derivatives is highlighted by recent FDA approvals of multiple cyclodextrin-containing formulations. Most of these formulations use HP-β-CD, but the list also contains products with native cyclodextrins, SBE-β-CD, M-β-CD, and HP-γ-CD. HP-β-CD is used in formulations for nearly all routes of administration except the nasal route [23]. By introducing various functional groups, modified cyclodextrins can significantly enhance drug stability as well as improve the aqueous solubility and dissolution performance of poorly water-soluble drugs. However, careful control of the degree of substitution is essential to achieve the desired physicochemical properties. Certain substituents may sterically occupy the cyclodextrin cavity, thereby competing with drug molecules for inclusion complex formation. Consequently, the selection of substituents that are unable to enter the cyclodextrin cavity is a critical consideration in the design of functionalized cyclodextrin derivatives [145].

The most widely used cyclodextrin derivative in pharmaceutical formulations over the last decade was HP-β-CD, followed by M-β-CD and SBE-β-CD. In the case of altretamine, complexation with HP-β-CD significantly enhanced dissolution compared with the free drug, particularly under intestinal pH conditions. Among the investigated systems, kneaded inclusion complexes exhibited higher dissolution rates than co-evaporated products, which was attributed to efficient drug entrapment within the cyclodextrin cavity and amorphization. When incorporated into tablets, the HP-β-CD complexes further enabled controlled and sustained release, where cyclodextrin primarily improved solubility while the lipid matrix modulated the release rate [89]. In contrast to altretamine, bicalutamide formed highly soluble inclusion complexes with HP-β-CD and SBE-β-CD, resulting in almost complete drug dissolution within 10 min. The rapid release was driven by increased solubility, strong complexation (particularly with HP-β-CD), and the amorphous nature of the complexes, demonstrating the suitability of cyclodextrin derivatives for immediate-release formulations [92]. The HP-β-CD inclusion complex of clausenidin exhibited pronounced sustained drug release, with only 3.4% released after 1 h and 29.4% after 12 h, followed by continuous release for more than five days at pH 7.4, in contrast to the physical mixture, from which clausenidin was completely released within 19 min [95]. In three independent studies, curcumin release from HP-β-CD-based systems was shown to be highly formulation-dependent. In simple HP-β-CD inclusion complexes, curcumin exhibited a very rapid and nearly complete release, reaching approximately 100% within minutes, in sharp contrast to free curcumin, which released less than 1% over 2 h [99]. When different preparation methods were compared, co-evaporation produced the highest cumulative release due to enhanced swelling and loading efficiency, while other methods yielded more moderate but still improved release profiles [97]. In more complex formulations, such as HP-β-CD-modified curcumin incorporated into gelatin-carrageenan microparticles, drug release became more sustained, with higher release at basic pH driven by polymer swelling and amorphization, demonstrating how additional formulation layers can shift curcumin delivery from rapid dissolution toward controlled release [98]. The same trend was also observed for formononetin after incorporation of its HP-β-CD inclusion complex into poly(lactic-co-glycolic acid) nanoparticles [115] and for paclitaxel after incorporation of its DM-β-CD inclusion complex into chitosan nanoparticles [122], as well as for HP-β-CD-based paclitaxel systems further encapsulated into poly-3-hydroxybutyrate [121]. When formulating nanosuspensions of oridonin-HP-β-CD inclusion complexes, dissolution was dramatically enhanced, reaching 100% within 2.5 min, compared with the incomplete release of initial oridonin and conventional suspensions [120]. Pomalidomide formed a 1:1 inclusion complex with SBE-β-CD, also resulting in a markedly faster and almost immediate drug release at both acidic and neutral pH [124]. Similarly, HP-β-CD complexation markedly accelerated sorafenib dissolution, reaching around 85% within 10 min, while the free drug showed slow and incomplete release (around 40% after 50 min) [127]. Dissolution studies showed that tamoxifen inclusion complexes with β-CD derivatives exhibited markedly faster and more complete drug release than the initial drug, with M-β-CD and HP-β-CD complexes achieving more than 90% dissolution within 15–30 min. This enhanced dissolution was successfully retained after incorporation of the complex with M-β-CD into tablets, which reached 100% drug release within 45 min, whereas the commercial tablet showed incomplete dissolution after 60 min [128]. The thymoquinone/HP-β-CD complex greatly improved drug release, with more than 52% released in 1 h and approximately 84% in 24 h, compared with around 5% and 9% for free drug, reflecting enhanced solubility and sustained dissolution [129]. The venetoclax/HP-β-CD complex showed enhanced solubility and sustained release, with 25% released at 1 h, 56% at 12 h, and 81.6% at 24 h, compared with 100% release of free venetoclax within 5 h [130]. These differences in drug release profiles, despite using the same cyclodextrin derivatives, are largely dictated by the formulation strategy and the physical state of the complex. Simple inclusion complexes or those with high swelling and amorphization tend to exhibit immediate release due to rapid solubilization of the drug. In contrast, incorporation into additional carriers can create diffusion barriers or slow-swelling matrices, resulting in sustained release, even though the same cyclodextrin derivative is present. Thus, the release behavior is controlled not only by cyclodextrin complexation, but also by the structural and compositional design of the final formulation.

During the last decade, epichlorohydrin-crosslinked β-CD polymers (Epi-β-CD) have also been explored for inclusion complex formation. In the case of camptothecin, grafting a tumor lytic peptide (ZH) onto the Epi-β-CD inclusion complex resulted in a sustained release profile (around 40% in 5 h and 70% in 24 h at pH 7.4), in contrast to faster release observed for free camptothecin and binary complexes [93]. Binary inclusion complexation of gefitinib with Epi-β-CD, in contrast, led to a markedly enhanced and rapid dissolution, with approximately four-fold higher drug release after 1 h compared with the pure drug, driven by amorphization and reduced surface tension [116]. Unlike gefitinib, selective grafting of D-glucose or D-maltose moieties onto β-CD resulted in pH-responsive and sustained release of DOX and MTX. After an initial burst, attributed to drug molecules bound to the carrier surface through electrostatic and hydrogen-bonding interactions, release became diffusion-controlled from the cyclodextrin cavity and dendritic structure. Higher drug release was observed under acidic conditions, while DOX exhibited a more prolonged release than MTX, highlighting the critical role of cyclodextrin functionalization and drug–carrier interactions in modulating release profiles [109,110]. DOX loaded into a β-CD dendrimer exhibited a pronounced pH-dependent and sustained release, with only 17% released within the first 3 h and prolonged release up to 72 h, compared with complete release of free DOX within 10 h. The enhanced control over drug release was attributed to diffusion within the dendritic network, stronger hydrophobic interactions, and slower dendrimer degradation, with higher release observed under acidic conditions [111]. In the case of MTX, a heptakis(2,3,6-tri-O-spacer-β-CD)-β-CD biodendrimer (β-CD-(spacer-β-CD)_21_) also provided a pronounced pH-dependent sustained release following an initial burst, with prolonged drug release over 72 h, particularly under acidic conditions [119]. Other drugs investigated for encapsulation with complex cyclodextrin derivatives over the past decade include docetaxel and erlotinib. In the case of docetaxel, inclusion complexation with β-CD derivatives resulted in distinct release behaviors depending on carrier design and triggering mechanisms. Alkylenediamine-modified β-CD inclusion complexes markedly enhanced docetaxel solubility and exhibited a biphasic release profile, characterized by an initial burst followed by sustained release over up to 48 h, largely independent of pH [105]. In contrast, a β-CD-docetaxel complex combined with maltogenic amylase enabled enzyme-responsive release, where it was negligible in the absence of the enzyme but accelerated proportionally with enzyme concentration [106]. These studies illustrate how β-CD systems can be tailored either for diffusion-controlled sustained release or for stimuli-responsive, on-demand drug release. Erlotinib has also been complexed with randomly methylated β-CD, resulting in a pronounced immediate-release behavior (around 75–80% released within 10 min compared with 23.5% for free erlotinib). This effect was mainly attributed to cyclodextrin-mediated solubilization, molecular-level particle size reduction, and drug amorphization [112].

An overview of the results reported during the last decade clearly shows that drug release from cyclodextrin-based systems does not follow a single universal pattern. Depending on the cyclodextrin derivative, type of modification, preparation method, and overall formulation design, inclusion complexes may exhibit either rapid dissolution or prolonged, controlled release. Simple binary complexes and highly amorphous systems often favor fast drug release through improved solubilization, whereas functionalized, polymeric, or dendritic cyclodextrins introduce additional diffusion barriers and stronger host–guest interactions, leading to sustained or stimuli-responsive release. Moreover, external factors such as pH, enzymatic activity, and carrier architecture further modulate release kinetics. These findings underscore that cyclodextrin complexation acts as a versatile formulation tool, while the final release behavior is governed by the combined effects of drug properties, cyclodextrin modification, and formulation strategy.

### 3.3. Mechanism of Inclusion Complex Formation

For optimal binding within the cyclodextrin cavity, the guest molecule must possess suitable functional groups. Since the interior of cyclodextrins is hydrophobic, they preferentially interact with hydrophobic organic molecules. Conversely, molecules containing polar or hydrophilic groups generally bind less effectively to the cyclodextrin cavity [140]. In most cases, van der Waals interactions are dominant stabilizing forces in cyclodextrin–guest complexes. Given that native cyclodextrins lack strongly ionizable groups under neutral conditions, hydrogen bonding represents the most significant intermolecular force in the formation of cyclodextrin complexes in solution [147]. Water molecules associated with the hydrophobic groups inside the cyclodextrin cavity lose approximately two hydrogen bonds, placing them in a thermodynamically unfavorable state. The displacement of these high-energy water molecules is a key driving force for the formation of inclusion complexes in aqueous solutions. When a guest drug molecule approaches the cyclodextrin, the hydrophobic regions of the molecule favor the expulsion of water molecules trapped within the hydrophobic cavity. These water molecules then move into the surrounding solution, where they can reform hydrogen bonds with other water molecules. This process lowers the overall energy of the system, as the water molecules return to a more stable state, thereby increasing the thermodynamic stability of the inclusion complex [148].

**Figure 9:**
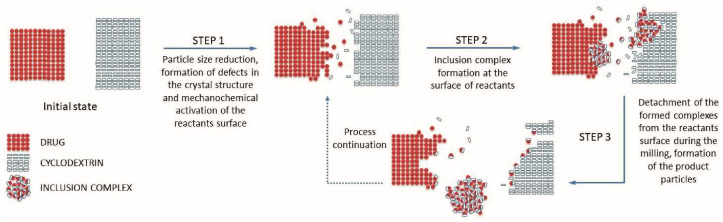
Potential mechanism of mechanochemical formation of inclusion complexes. Reproduced from M. Jug et al. [150]; licensed under CC BY.

Mechanochemical synthesis has emerged as an increasingly important approach for preparing cyclodextrin-based inclusion complexes in the solid state, using various types of mills (planetary ball mills, vibrational mills, cryogenic mills) [149]. The popularity of mechanochemical synthesis via grinding stems from its effectiveness in promoting both coordination and non-covalent interactions, such as hydrogen bonds, halogen bonds, and π···π aromatic stacking. Although the detailed mechanism of inclusion complex formation during the co-grinding of drug and cyclodextrin mixtures is not fully understood, a possible scenario is illustrated in Figure 9 [150]. It is based on a general three-step mechanism proposed for mechanochemical reactions, while also considering other processes that occur during the mechanochemical activation of the drug through milling. When a mixture of drug and cyclodextrin is subjected to milling, it receives mechanical impulses each time the material is trapped between colliding grinding bodies or between a body and the mill wall. If such impacts are sufficiently intense, they result in localized, near-adiabatic energy accumulation, leading to the formation of a metastable structure [151]. Additionally, localized stresses within crystalline regions induce fracture, reducing particle size and increasing the total surface area available for solid-state interactions between the drug and cyclodextrin (step 1) [150,152]. Sustained energy input leads to amorphization of the crystalline materials in the treated mixture, typically initiating at a thin surface layer and then propagating inward, producing an activated material. Reactions of the activated materials at the surfaces of the drug and cyclodextrin particles may involve several intermediate stages, such as the formation of a solid dispersion, which gradually transforms into a genuine solid-state inclusion complex through molecular diffusion (step 2). As milling progresses, the synthesized inclusion complex can separate from the drug or cyclodextrin particles, generating discrete particles and exposing new surfaces for continued reaction (step 3) [150]. Finally, milling provides intensive mixing and homogenization of the reactants, further promoting solid-state interactions [153]. A mechanistic understanding of inclusion complex formation is essential for the rational design of cyclodextrin-based systems with predictable drug release behavior.

## 4. Metal–Organic Frameworks

**Figure 10:**
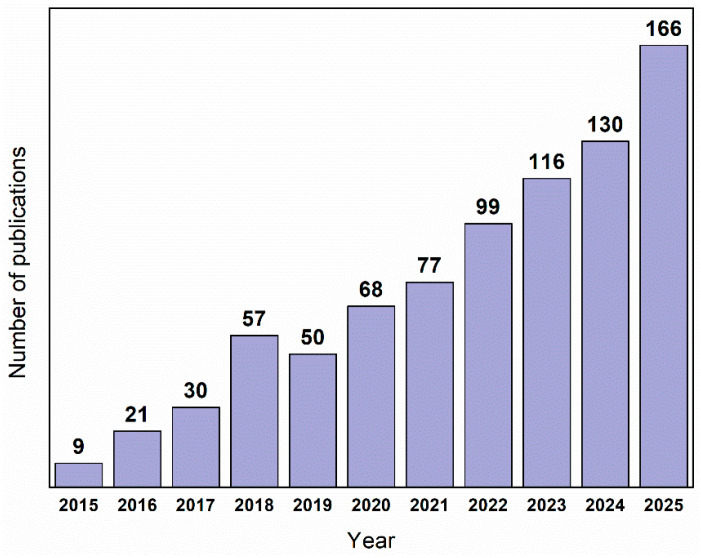
Number of publications indexed in the Scopus database between 2015 and 2025 related to the use of MOFs as carriers in anticancer drug formulation, obtained using the keywords “metal–organic frameworks” and “anticancer drug.”

A pronounced and consistent upward trend in publications was observed during the Scopus and PubMed literature search using the keywords “metal–organic frameworks” and “anticancer drug.” In total, 823 publications were identified in this category (Figure 10). Of these, 658 were original research articles, 136 were review papers, and 10 were book chapters. In addition, 4 conference papers, 2 errata, 1 short survey, and 1 editorial were identified, while 11 publications were retracted. The observed linear growth indicates a steadily increasing scientific interest in these systems, highlighting their growing relevance within nanomedicine and advanced drug delivery. The growing number of publications reflects ongoing efforts to develop innovative carrier platforms aimed at addressing key limitations of conventional anticancer therapies. In this context, MOF-based formulations have attracted considerable attention due to their potential to enable controlled and sustained drug release, as well as to improve pharmacokinetic and pharmacodynamic profiles of anticancer agents. By facilitating more precise control over drug release behavior, these systems may contribute to reduced dosing frequency, lower off-target toxicity, and improved therapeutic efficacy. Overall, the continuous increase in publication output underscores the significance of MOFs as an emerging and rapidly evolving research area in anticancer drug delivery, warranting comprehensive analysis and critical discussion in the following sections of this review.

One of the earliest attempts to incorporate an anticancer drug into nanoscale coordination polymers dates back to 2008, when Rieter et al. constructed a system based on Tb^3+^ ions and disuccinatocisplatin, subsequently encapsulated within amorphous silica shells. This work represents one of the earliest examples of metal–organic nanomaterials designed for controlled anticancer drug delivery, in which a platinum(IV) prodrug was incorporated as a structural component of the coordination polymer [154]. Due to their exceptional porosity, high surface area, and high chemical stability, MOFs were traditionally explored primarily for applications such as gas storage, catalysis, sensing, water treatment, and membrane separation [15]. In recent years, MOFs have demonstrated significant potential in drug delivery and have made notable progress in pharmaceutical research, driven by their exceptionally high loading capacity and the ability to release therapeutics in response to specific stimuli [155]. The choice of framework composition and drug-loading approach can significantly enhance the therapeutic outcome of MOF-based systems. Synergy may arise from the incorporation of more than one therapeutic agent within a single MOF or from the concurrent release of the drug along with metal ions or organic linkers derived from the framework [28]. While MOF-based drug delivery systems are not yet clinically approved, ongoing advances in framework engineering, surface modification, and biocompatible compositions have improved their performance, sustaining strong interest in their biomedical and translational potential [156]. In this context, Table 3 summarizes MOF platforms investigated for anticancer drug delivery between 2015 and 2025, together with the corresponding therapeutic agents and selected design parameters. In contrast to the examples discussed for polymeric solid dispersions and cyclodextrin-based inclusion complexes, only representative examples are presented here for each investigated anticancer agent, given the large number of available studies. As synthetic protocols for many MOF architectures are already well established, Table 3 therefore focuses on the drug-loading strategy rather than on framework preparation details. Here, the loading strategy distinguishes between in situ loading, in which the anticancer agent is present during MOF formation, and post-synthetic loading, where the drug is introduced into a preformed framework.

As summarized in Table 3, MOF-based anticancer drug delivery systems investigated between 2015 and 2025 include a wide range of carrier structures, anticancer agents, and drug-loading strategies. Zinc-based ZIF structures and zirconium-based UiO-type frameworks are among the most frequently used carriers, reflecting their chemical stability, tunable porosity, and physiological compatibility. Across the reviewed studies, post-synthetic loading emerged as the dominant approach for drug incorporation, while one-pot encapsulation remained comparatively less frequent, likely reflecting its greater potential to interfere with MOF crystallization, framework purity, and reproducibility when the drug is present during synthesis [225]. It is important to emphasize that, despite the increasing number of studies on MOF-based materials, many of the reported systems are not primarily intended for drug delivery. Many studies instead investigate MOFs for cancer-related sensing, imaging, or theranostic applications, such as electrochemical detection of anticancer drugs in biological samples, fluorescence-based monitoring of apoptosis-associated biomarkers, and imaging-guided assessment of anticancer activity [226]. While these contributions are scientifically valuable to oncology research, they fall outside the scope of controlled drug delivery and were therefore excluded from the present comparison.

Earlier MOF-based drug delivery studies relied predominantly on a limited set of model chemotherapeutics, most notably DOX and 5-FU. These compounds were favored due to their well-characterized physicochemical properties and straightforward quantification in release studies. As reflected in Table 3, DOX remains one of the most extensively investigated anticancer agents and continues to be employed in both early proof-of-concept studies aimed at demonstrating drug loading and release feasibility, as well as more recent multifunctional MOF platforms. A broader range of anticancer agents has been explored only in more recent studies, particularly since 2021, reflecting advances in MOF synthesis, characterization, and drug-loading strategies. When considered alongside the formulation approaches discussed in the preceding sections, MOF-based drug delivery systems have often demonstrated more prolonged drug release profiles than amorphous solid dispersions or cyclodextrin inclusion complexes, extending from hours to days or even weeks. This observation underscores the potential of MOFs to overcome key limitations associated with conventional formulation strategies by enabling prolonged drug retention within the carrier structure and controlled release behavior.

These findings highlight that MOF-based anticancer drug delivery systems represent a highly versatile and complex formulation platform, in which drug selection, framework composition, and loading strategy collectively govern release kinetics and therapeutic performance. From the broad range of reported systems, several particularly relevant categories were identified and are discussed in detail in the following subsections. Among currently investigated delivery platforms, MOFs arguably offer the highest degree of structural control over drug release.

### 4.1. Stimuli-Responsive MOF Carriers for Controlled Anticancer Drug Release

**Figure 11:**
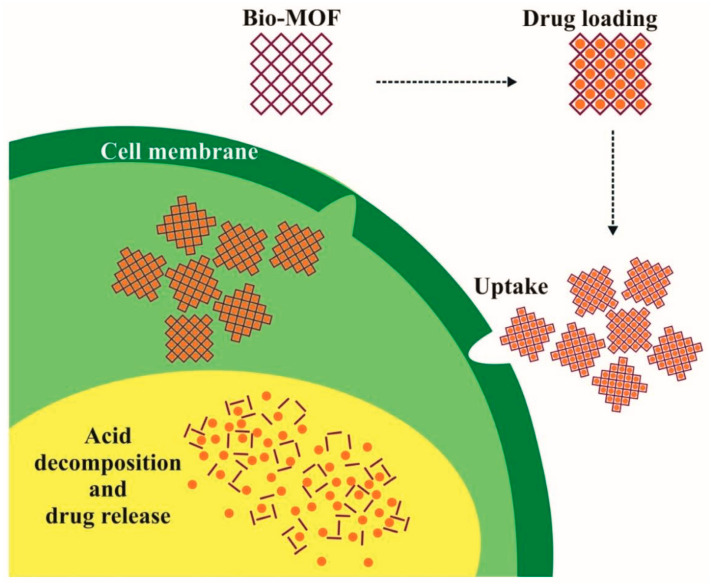
Mechanism of pH-responsive disassembly in MOF-based drug carriers. Reproduced from C. Păun et al. [230]; licensed under CC BY.

Stimuli-responsive drug delivery systems are designed to release therapeutic agents in response to specific internal or external stimuli. Upon administration, these systems undergo controlled physicochemical or structural changes when exposed to defined triggers associated with the tumor tissue, thereby promoting the release of the incorporated drug [227]. Internal stimuli include pH gradients, biomolecules, and redox-related processes characteristic of the tumor microenvironment, which can induce carrier destabilization, framework degradation, or disruption of drug–carrier interactions, thereby promoting drug release. In contrast, external stimuli include light irradiation, magnetic fields, ultrasound, and temperature changes, which enable on-demand activation of drug release through externally controllable physical inputs [228]. Among stimuli-responsive MOF carriers, pH-responsive systems are the most extensively studied, owing to the mildly acidic tumor microenvironment and the inherent sensitivity of coordination bonds within MOF structures to pH variations. Consequently, a wide range of pH-responsive MOF platforms has been reported for controlled anticancer drug delivery (Figure 11) [229]. Metabolic dysregulation in tumor cells leads to elevated levels of reactive oxygen species (ROS) and reducing agents such as glutathione (GSH) within the tumor microenvironment. Certain MOFs are designed to respond to these redox conditions, enabling controlled drug release triggered by oxidative or reductive stimuli. MOFs have also been reported to exhibit enzyme-, glucose-, ATP-, H_2_S-, and ion-responsive drug release [227].

Zeolitic imidazolate frameworks have frequently been investigated as stimulus-responsive platforms owing to the tunable stability of their coordination networks. A representative example is the DOX-loaded ZIF-8 system, which exhibited minimal drug release under physiological conditions but substantially accelerated release in acidic environments as a consequence of framework destabilization. In contrast, the structurally more robust ZIF-7 displayed negligible responsiveness under the same conditions, emphasizing the critical role of framework stability in regulating drug release. This effect was further enhanced in membrane-mimicking environments, where electrostatic interactions between DOX and negatively charged micelles or liposomes facilitated diffusion from the framework. These results indicate that both structural characteristics of the framework and interactions with biologically relevant interfaces play a decisive role in the design of stimulus-responsive MOF-based drug delivery systems [186]. Zirconium-based MOFs have also demonstrated pH-dependent release behavior. For example, the porphyrin-containing framework PCN-222 enabled sustained release of oridonin for over seven days in vitro. Drug release was more pronounced under mildly acidic conditions (about 86% at pH 5.5) than at near-neutral pH (about 63% at pH 7.2), consistent with diffusion-controlled release. The carrier further exhibited good biocompatibility and enhanced cytotoxicity against HepG2 cells compared with the free drug, supporting its potential for tumor-targeted delivery [210]. Similarly, the iron-based MIL-53 (Fe) exhibited comparable release behavior and biological performance [211]. In contrast, oridonin@MOF-5 showed release behavior that was largely independent of pH, with similar cumulative release (about 87%) observed across the tested conditions [212]. In the case of quercetin, UiO-66 and its functionalized analogs also demonstrated prolonged release, with the –NO_2_-modified framework showing higher drug release under acidic conditions (about 94%) compared with physiological pH (about 72%) [217].

A redox-responsive MOF-Zr (DTBA) was developed to enable GSH-triggered drug release. Curcumin-loaded nanoparticles achieved significantly higher drug release in the presence of GSH (about 87% within 22 h) than under non-reducing conditions (about 50%), while the combination of reductive and mildly acidic environments resulted in almost complete release within 5 h. Enhanced anticancer activity was also confirmed in both in vitro and in vivo studies [177]. An ATP-responsive MOF platform has also been described, where nucleic acid-capped nanoparticles remained sealed until exposure to elevated ATP levels characteristic of cancer cells. ATP binding to the aptamer induced framework opening and triggered DOX release, while drug release remained minimal in its absence. In this platform, ATP responsiveness was mainly associated with elevated intracellular ATP levels in cancer cells rather than extracellular ATP in the tumor microenvironment alone. The authors used 25 mM ATP in release experiments to obtain a clear in vitro response, while noting that ATP concentrations of 10 mM or higher have been reported in cancer cells. DOX release increased with increasing ATP concentration, supporting the ATP-dependent gating mechanism. Selectivity was further enhanced by the AS1411 aptamer, which promoted preferential uptake by MDA-MB-231 breast cancer cells over normal MCF-10A epithelial breast cells and contributed to the high cytotoxic efficacy observed toward cancer cells [196].

Dual-responsive systems have also been reported, combining multiple triggers to further regulate drug release. For example, MTX-loaded zinc-based frameworks ZJU-64 and ZJU-64-CH_3_ exhibited sustained release under physiological conditions, while elevated temperature further accelerated drug release by weakening host–guest interactions [206]. A similar dual-responsive behavior was observed for MTX-encapsulated Zn-TBDA, where drug release was markedly accelerated under tumor-like conditions. After 48 h, only about 43% of the drug was released at physiological pH and temperature, whereas significantly higher release occurred at lower pH and 42 °C, indicating that the combined effect of acidity and hyperthermia can promote more efficient drug delivery [207]. Dual responsiveness was further demonstrated in the Zn-GA framework, where MTX release increased with decreasing pH and increasing temperature. Only about 43% of the drug was released under physiological conditions after 72 h, compared with about 68% in acidic media, with an additional rise observed at 42 °C. The enhanced release was attributed to partial structural destabilization, confirming the potential of such systems for controlled delivery in tumor-like environments [208]. Overall, these findings indicate that multi-stimuli-responsive MOFs provide an additional level of control over drug release, supporting more selective delivery while maintaining limited drug release under physiological conditions. Such systems therefore represent a promising strategy for supporting more precise drug delivery and therapeutic efficiency of anticancer therapy.

### 4.2. Multifunctional and Theranostic MOF Hybrid Systems

**Figure 12:**
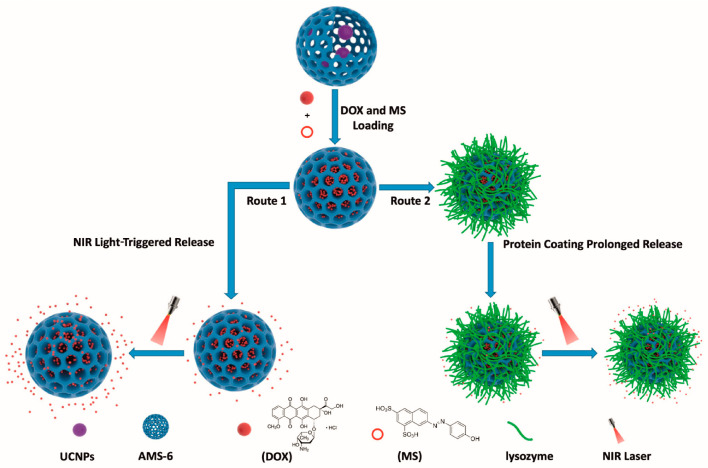
Representative example of a multifunctional UCNP-MOF hybrid system enabling imaging-guided and NIR-triggered anticancer drug delivery. Reproduced from Y. Huang et al. [234]; licensed under CC BY.

In recent years, growing attention has shifted toward multifunctional MOF-based hybrid systems that extend the role of MOFs beyond drug delivery alone. By incorporating additional nanomaterials into the framework, such platforms can combine controlled drug delivery with imaging capabilities or externally triggered therapeutic effects, with the potential to improve therapeutic outcomes. Theranostic systems integrate therapeutic and diagnostic functions within a single platform, enabling simultaneous treatment and imaging-based monitoring of disease progression and therapeutic response [231]. Among the commonly explored strategies is the incorporation of upconversion nanoparticles (UCNPs), which convert near-infrared (NIR) light into higher-energy visible emission, thereby enabling optical imaging and externally triggered therapeutic effects (Figure 12). The use of NIR irradiation, characterized by deeper tissue penetration and minimal photodamage, further highlights the suitability of UCNP-containing systems for anticancer applications [232]. In addition, the integration of metal nanoparticles into MOF carriers has been widely investigated to introduce magnetic, photothermal, or catalytic functionalities [233]. Together, these design strategies enable the combination of imaging, stimulus-responsive therapy, and controlled drug delivery within a single theranostic platform.

UCNP-based MOF hybrids are emerging as promising platforms for targeted anticancer drug delivery with integrated imaging capabilities. Core–shell systems constructed by growing folic acid-functionalized MOF layers on UCNP surfaces have enabled the encapsulation of chemotherapeutics such as 5-FU and DOX while preserving luminescent properties for imaging. Drug release from these carriers was significantly enhanced under mildly acidic conditions characteristic of the tumor microenvironment. For example, a 5-FU-loaded UCNP@ZIF-8 platform released approximately 82% of the drug at pH 5.5 compared with about 41% at physiological pH within 24 h, indicating selective release behavior. A comparable architecture based on UCNP@UiO-66-NH_2_ demonstrated similar responsiveness, with DOX release reaching about 72% at pH 5.5 versus about 40% at pH 7.4 over the same period. Folate receptor-mediated uptake further enhanced cytotoxicity in cancer cells, while the UCNP core supported simultaneous imaging. Such systems highlight the potential of UCNP-integrated MOF hybrids as advanced theranostic platforms for precise and controlled anticancer drug delivery [157,193].

Metal nanoparticle-integrated MOF hybrids represent an important class of multifunctional carriers that combine controlled drug delivery with imaging and externally triggered therapeutic effects. A representative example is the CoFe_2_O_4_@PDA@ZIF-8 core–shell nanocomposite designed for the co-delivery of DOX and camptothecin. The system exhibited minimal drug release at physiological pH, whereas acidic conditions promoted significantly higher release together with a sequential pattern in which camptothecin was released first, followed by DOX after approximately 12 h. Upon NIR irradiation, rapid drug release was induced through photothermal disruption of the structure, thereby enabling simultaneous chemotherapy and photothermal therapy. The platform demonstrated low toxicity and a pronounced synergistic antitumor effect in both cell-based and tumor-bearing mouse models [170]. Magnetic hybrids have also been explored to integrate imaging with controlled delivery. Fe_3_O_4_@IRMOF-3 nanoparticles functionalized with folic acid and a fluorescent probe enabled targeted delivery of paclitaxel while simultaneously providing magnetic resonance and fluorescence imaging capabilities. The system showed sustained drug release under physiological conditions and good cytocompatibility, supporting its potential as an imaging-guided delivery platform [214]. A related strategy involved embedding magnetic MIL-53 nanoparticles within poly(acrylic acid)-grafted chitosan/polyurethane core–shell nanofibers for the co-delivery of temozolomide and paclitaxel. The carriers provided gradual drug release without an initial burst, while both acidic conditions and magnetically induced hyperthermia accelerated drug release. This effect was attributed to increased matrix swelling, enhanced polymer mobility, and greater porosity, ultimately leading to higher apoptosis rates in glioblastoma cells during combined chemotherapy and hyperthermia treatment [215]. Lastly, metal incorporation has also been used to modulate tamoxifen release behavior. Silver-impregnated MOF-808 nanocomposites enabled sustained drug release, reaching about 77% within 36 h, slightly higher than the non-modified framework. The presence of silver nanoparticles likely reduced drug–framework interactions, facilitating diffusion while preserving controlled release characteristics [220].

Taken together, these studies demonstrate that integrating magnetic, metallic, or optically active components into MOF structures can substantially broaden their functionality, including multimodal imaging, externally triggered therapy, and combination treatment strategies, while maintaining regulated drug release profiles. Such hybrid systems therefore represent an important direction for the development of advanced multifunctional and theranostic platforms.

### 4.3. Cyclodextrin-Based MOFs for Anticancer Drug Delivery

**Figure 13:**
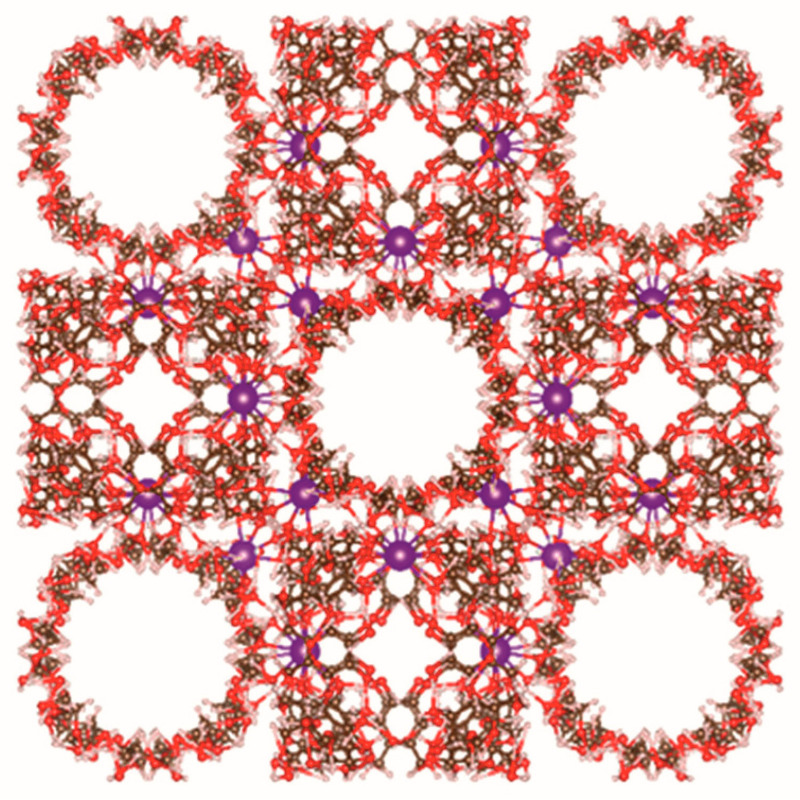
Schematic representation of the porous γ-CD-based MOF architecture. Hydrogen, carbon, oxygen, nitrogen, and potassium atoms are depicted in pink, brown, red, blue, and purple, respectively. Adapted from C. Watson et al. [237]; licensed under CC BY.

Safety considerations associated with synthetic organic linkers have increasingly raised concerns regarding the use of conventional MOFs in biomedical applications. Consequently, cyclodextrins have emerged as attractive building blocks for MOF construction, enabling the development of more biocompatible porous systems [235]. Within the field of anticancer drug delivery, CD-MOFs have attracted particular interest due to their ability to accommodate structurally diverse therapeutic agents while supporting controlled release profiles. Their unique framework architecture facilitates high drug loading while potentially improving the solubility and stability of poorly water-soluble chemotherapeutics, making CD-MOFs promising carriers for enhancing therapeutic efficiency [236]. In this way, CD-MOFs establish a hybrid platform that unites the complementary features of both cyclodextrins and MOFs, opening new opportunities for the design of more effective anticancer drug delivery systems (Figure 13).

The potential of CD-MOF-based systems for anticancer drug delivery has been demonstrated using β-CD-MOFs functionalized with glutamine or coated with gelatin for curcumin delivery. These systems showed pH-dependent release, with markedly higher release at pH 5.5 than at pH 7.4, while glutamine functionalization and gelatin coating reduced burst release and supported a more sustained profile [178]. A pH-responsive co-delivery system was also reported using magnetic lactose-modified ZIF-8 crosslinked with β-CD for DOX and curcumin delivery to MCF-7 cells. The carrier released both drugs preferentially under acidic conditions, following Korsmeyer–Peppas kinetics and predominantly Fickian diffusion [179]. Crosslinked CD-MOFs have also been adapted for lung-targeted DOX delivery. He et al. designed a nanoplatform based on RGD-functionalized crosslinked γ-CD-MOF nanoparticles conjugated with low-molecular-weight heparin (RGD-CDF-LMWH), where RGD refers to the tumor-targeting arginine-glycine-aspartic acid peptide motif. DOX release from this system was GSH-sensitive, increasing from minimal release at pH 7.4 to approximately 50% and 80% in the presence of 1 and 10 mM GSH, respectively. This system preferentially accumulated in lung tumors and significantly reduced metastatic burden without evident systemic toxicity [197]. A representative example is the crosslinked γ-CD-based MOF (TPT@CL-MOF) developed by Xiong et al. for the delivery of topotecan, a drug whose clinical use is limited by rapid hydrolysis of its active lactone form under physiological conditions [223]. Encapsulation within the porous cyclodextrin matrix enabled sustained drug release, markedly reducing the initial burst effect observed for the free drug and extending release to approximately 48 h. The carrier also enhanced chemical stability, increasing the hydrolysis half-life of the lactone form from about 0.93 to 22.05 h, which was attributed to host–guest interactions that shield the drug from the aqueous environment. Biological evaluation further supported the therapeutic relevance of this platform. The formulation exhibited good cytocompatibility while maintaining pronounced anticancer activity in vitro, including inhibition of melanoma cell migration and invasion. Fluorescence-based biodistribution studies revealed preferential accumulation in the lungs following intravenous administration, highlighting its suitability for pulmonary drug delivery. In a metastatic lung tumor model, TPT@CL-MOF significantly reduced tumor burden and achieved comparable therapeutic efficacy at a substantially lower drug dose without evident systemic toxicity. CD-MOFs have also been investigated for improving the oral delivery of triptolide in hepatocellular carcinoma. Li et al. prepared triptolide-loaded γ-CD-MOFs to improve the solubility, bioavailability, and anticancer efficacy of triptolide. Loading into the CD-MOF increased the equilibrium solubility of triptolide approximately 9.5-fold compared with the free drug, likely due to conversion from the crystalline to the amorphous state. In vitro release studies showed that TPL@CD-MOF released triptolide more slowly than the initial drug under simulated gastrointestinal conditions, suggesting that drug diffusion was hindered after encapsulation within the framework. This slower release was accompanied by improved bioavailability and a modest but statistically significant enhancement of antitumor efficacy both in vitro and in vivo [224].

Despite growing interest, studies specifically addressing CD-MOF-based systems for anticancer drug release remain relatively limited. Nevertheless, crosslinked cyclodextrin frameworks illustrate how combining host–guest chemistry with porous architectures may help address key challenges associated with drug instability and undesired release profiles. By integrating the established safety profile of cyclodextrins with the structural tunability of MOFs, CD-MOFs may represent an important step toward clinically translatable porous drug delivery systems.

## 5. Challenges and Future Perspectives in Anticancer Drug Delivery

Over the past decade, drug delivery technologies have progressively evolved from conventional solubility-enhancing approaches toward increasingly sophisticated and multifunctional platforms. Polymeric solid dispersions and cyclodextrin-based inclusion complexes represent well-established formulation strategies, with several products already translated into clinical use [141,238]. Their regulatory acceptance, proven safety profiles, and manufacturing feasibility clearly distinguish them from emerging material classes and confirm their practical relevance in contemporary pharmaceutical development. By contrast, MOF-based systems remain mainly preclinical. Nevertheless, they provide useful experimental models for examining advanced release-control concepts, including stimuli-responsive and multifunctional delivery. At the same time, all three platforms differ substantially in their ability to regulate drug release, which remains a central parameter for improving therapeutic efficacy and safety. To provide a concise comparison of these formulation strategies, their key advantages, limitations, release characteristics, stability considerations, and translational applicability are summarized in Table 4.

Beyond release performance, the translational potential of these platforms is also determined by stability, safety, reproducibility, scalable manufacturing, and regulatory acceptability. These factors are particularly important for MOF-based formulations, where structural tunability and stimuli-responsive behavior must be balanced against biological stability and long-term safety requirements. Polymeric solid dispersions remain among the most reliable approaches for enhancing the dissolution and oral bioavailability of poorly water-soluble anticancer drugs. However, their primary function is rapid solubilization rather than precise regulation of drug release. In many cases, drug release is primarily governed by hydration and dissolution of the polymer matrix, which often results in rapid or moderately prolonged release rather than tightly regulated kinetics. Although advances in polymer selection, ternary formulations, and self-emulsifying systems have enabled partial modulation of release kinetics, achieving truly sustained and predictable drug release remains challenging. Future research should therefore prioritize improving control over release behavior while maintaining physical stability and scalable manufacturing. Cyclodextrin-based carriers have progressed from classical solubilizing excipients to structurally adaptable delivery platforms capable of modulating drug release. Simple inclusion complexes frequently promote fast drug dissolution due to enhanced wettability and amorphization, yet more advanced architectures such as ternary complexes, nanosponges, hydrogels, and polymer-integrated systems can introduce diffusion barriers that enable sustained or stimuli-responsive release. Moving forward, optimizing the balance between complex stability and efficient drug release while maintaining formulation simplicity will be essential for maximizing their therapeutic impact.

In contrast, MOFs represent a comparatively young yet rapidly expanding field in anticancer drug delivery and demonstrate pronounced capabilities for controlled drug release, which can extend from days to even weeks. Their exceptional porosity, tunable architecture, and capacity for stimuli-responsive behavior position them among the most promising next-generation carriers. However, MOF-based formulations have not yet achieved clinical translation, and several specific barriers remain unresolved. Long-term stability in biological fluids must be demonstrated, since premature framework degradation may cause uncontrolled release, whereas excessive stability may hinder biodegradation and clearance. In addition, acceptable toxicity profiles must be established not only for the intact carrier, but also for released metal ions, organic linkers, and degradation products. Reproducible synthesis with consistent particle size, morphology, crystallinity, surface chemistry, and drug loading is also essential, particularly for scale-up and regulatory evaluation. Addressing these issues through systematic stability, degradation, toxicity, pharmacokinetic, and manufacturing studies will be critical for future development. Encouragingly, the increasing use of biocompatible metals and hybrid architectures suggests a clear trajectory toward more clinically acceptable designs.

**Figure 14:**
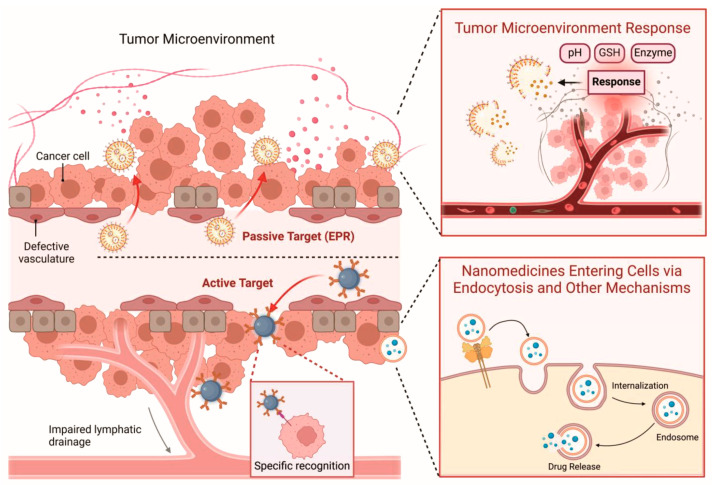
Targeting mechanisms supporting site-selective drug release in the tumor microenvironment. Reproduced from S. Fan et al. [243]; licensed under CC BY.

A broader perspective suggests that the future of anticancer drug delivery is unlikely to depend on a single dominant platform, but rather on the strategic integration of complementary technologies. This perspective also includes passive and active targeting, tumor microenvironment-responsive release, and cellular internalization as complementary mechanisms supporting site-selective anticancer drug delivery (Figure 14). Well-established systems offer proven safety profiles and manufacturing feasibility, while emerging nanostructured carriers enable a higher degree of control over drug release and therapeutic performance. Continued progress in this field will therefore require close alignment between material innovation, pharmaceutical practicality, and predictive formulation design. In this context, artificial intelligence and machine learning are emerging as useful tools for supporting the rational design of anticancer drug delivery systems. These approaches may assist in predicting drug–carrier compatibility, selecting suitable polymers, cyclodextrins, or MOF structures, optimizing formulation variables, and modeling release kinetics. Data-driven approaches may also accelerate the development of stimuli-responsive systems, targeted carriers, and multifunctional hybrid nanocarriers by identifying relationships between carrier composition, physicochemical properties, release behavior, and biological performance. Particular attention should nevertheless remain directed toward biocompatibility, scalable production, and predictable in vivo behavior alongside therapeutic efficacy. Advancing these aspects will be essential for translating promising formulation concepts into clinically relevant treatments and for supporting the transition from experimental carriers to robust drug delivery platforms capable of addressing the complex demands of modern oncology.

## 6. Conclusions

Substantial progress has been achieved in the development of advanced anticancer drug delivery formulations over the past decade, aimed at improving the solubility, stability, and release behavior of therapeutic agents. Polymeric solid dispersions and cyclodextrin-based inclusion complexes remain clinically relevant platforms, offering proven manufacturability, regulatory acceptance, and reliable enhancement of drug bioavailability. In parallel, MOFs have emerged as highly versatile carriers characterized by high drug-loading capacity and the ability to enable prolonged, stimuli-responsive release. Their structural tunability and multifunctional potential position them among the most promising next-generation delivery systems, although important challenges related to safety, biodegradability, and clinical translation still remain. Taken together, the comparative analysis presented in this review highlights that each formulation strategy addresses distinct therapeutic needs. Established technologies provide translational stability, whereas emerging nanostructured platforms provide greater control over drug release and functionality. Future progress in anticancer drug delivery will likely depend on balancing these attributes, with particular emphasis on biocompatibility, scalable production, and predictable in vivo behavior to enable the transition from innovative materials to clinically applicable therapies.

## Figures and Tables

**Table 1:** Anticancer agents and polymers used in solid dispersion systems (2015–2025).

Anticancer Agent	Anticancer Activity	Cancer Relevance	Polymer	Preparation Method	Release Behavior	Ref.
Andrographolide	Promoting cancer cell apoptosis, inhibiting proliferation and invasion, arresting the cell cycle, and suppressing angiogenesis.	Broad anticancer research context	Eudragit^®^ S100	Solvent evaporation	pH-responsive rapid release	[33]
Apalutamide	Blocking the effects of testosterone to slow or stop the growth of cancer cells.	Prostate cancer	PVP-K30, HPMCAS, Soluplus^®^, Eudragit^®^ L100-55	Solvent evaporation	Amorphization-, polymer-, and surfactant-dependent enhanced release	[34]
Apigenin	Modulation of signaling pathways involved in cell cycle, angiogenesis, and metastasis.	Broad anticancer research context	Chitosan	Spray drying	Initial burst followed by sustained anomalous release	[35]
Bicalutamide	Inhibition of androgen receptor signaling.	Prostate cancer	PEG 6000, Poloxamer 407	Mechanochemical activation	Enhanced release via improved wettability, solubilization, and reduced crystallinity	[36]
Curcumin	Inhibition of cancer cell proliferation via COX-2 and NF-κB pathway suppression.	Broad anticancer and chemopreventive research context	Eudragit^®^ EPO, PEG 6000, PVP K-30, HPMC 606 + Tween 80	Solvent evaporation, spray drying	Rapid release; enhanced release from ternary systems	[37,38,39]
Dasatinib	Targeting multiple kinases, inhibition of tumor growth and metastasis.	Leukemias and selected solid tumors	PVP-K30	Mechanochemical activation	Enhanced release governed by amorphization efficiency	[40,41]
Docetaxel	Stabilization of microtubules leading to mitotic arrest and apoptosis.	Breast, lung, prostate, gastric, and head and neck cancers	PVP K-30, HPMC E5, PEG 4000, PEG 10000, Eudragit^®^ EPO, Eudragit^®^ L100 + SDS	Freeze drying	Surfactant-assisted enhanced release	[42]
Elacridar hydrochloride	Enhancement of intracellular accumulation of chemotherapeutic agents by blocking drug efflux, potentially overcoming multidrug resistance in cancer cells.	Multidrug resistance modulation and chemosensitization	PVP K-30, PVP/VA 64, Soluplus^®^ + SDS	Freeze drying, spray drying	Polymer-dependent micellar release; supersaturation-enhanced release	[43]
Erlotinib hydrochloride	Targeting the epidermal growth factor receptor (EGFR).	Non-small cell lung cancer and pancreatic cancer	PVP K-30, PEG 4000, PVP K-30:PEG 4000 1:1	Solvent evaporation	Improved dispersibility from amorphization; enhanced solubility in ternary dispersion	[44]
Flutamide	Inhibition of androgen receptor signaling.	Prostate cancer	PVP K-90, HPMC, Eudragit^®^ EPO, PEG 8000	Solvent evaporation	Polymer-dependent enhanced release from amorphous dispersions	[45]
Ibrutinib	Inhibition of BTK signaling, suppression of the proliferation of malignant B cells.	B-cell malignancies	Poloxamer (P188, P237, P338, P407), Eudragit^®^ FS100	Spray drying, hot-melt extrusion	Rapid and nearly complete release in acidic media	[46,47]
Kaempferol	Induction of apoptosis, inhibition of cell proliferation, and modulation of oxidative stress and signaling pathways in various cancer cells.	Broad anticancer research context	Poloxamer 407	Solvent evaporation, fusion method	Rapid complete release from poloxamer-based dispersion	[48]
Lapatinib ditosylate	Blocking HER2 activity to suppress tumor cell proliferation.	HER2-positive breast cancer	PVP K-30, HPMC E5, L-HPC + SDS PVP K-90	Freeze drying, solvent evaporation	pH-responsive release enhanced by amorphization and nanosizing	[49,50]
Methotrexate	Inhibition of dihydrofolate reductase, blocking DNA synthesis and cell proliferation, particularly in rapidly dividing cancer cells.	Leukemias, lymphomas, breast cancer, osteosarcoma, and other cancers	PVP K-30 + TPGS	Emulsification	Sustained Fickian diffusion-controlled release	[51]
Naringin	Suppression of tumor growth, induction of apoptosis, and cell cycle arrest.	Broad anticancer research context	Kollidon^®^ VA 64, Kollidon^®^ VA 30, Soluplus^®^, Poloxamer 188	Freeze drying	Rapid release from amorphous polymeric dispersions	[52]
Paclitaxel	Stabilization of microtubules, inhibition of cell division, and induction of apoptosis in rapidly dividing cancer cells.	Breast, ovarian, lung, pancreatic, and Kaposi sarcoma	MPEG-PCL copolymer	Solvent evaporation	Sustained micellar release	[53]
Tacrolimus	Calcineurin inhibitor that modulates the immune response with potential anticancer effects.	Immunomodulatory and combination anticancer research context	MPEG-PCL copolymer	Solvent evaporation	Sustained micellar release	[53]
Telaprevir	Inhibition of proteasome activity and induction of apoptosis in cancer cells.	Repurposing and investigational anticancer context	PVP K-30, PEG 6000, HPMC	Mechanochemical activation	Polymer-dependent enhanced release via amorphization	[54]

**Table 2:** Anticancer agents and cyclodextrins used in inclusion complex systems (2015–2025).

Anticancer Agent	Anticancer Activity	Cancer Relevance	Cyclodextrin	Preparation Method	Release Behavior	Ref.
5-fluorouracil	Interference with the metabolism of DNA and RNA, leading to cell death.	Colorectal, gastric, pancreatic, breast, and head and neck cancers	β-CD, HP-β-CD	Freeze drying, in solution	Prolonged and controlled release	[87,88]
Altretamine	Used for recurrent ovarian cancer after platinum-based chemotherapy, working by damaging DNA in fast-dividing cells.	Ovarian cancer	HP-β-CD	Kneading, solvent evaporation	Sustained release	[89]
Amlodipine	Inhibition of cancer cell proliferation, promotion of apoptosis, and suppression of migration and invasion.	Repurposing and anticancer research context	β-CD, HP-β-CD, M-β-CD, SBE-β-CD	In solution, co-precipitation	Initial burst followed by diffusion-controlled release	[90]
Amygdalin	Arresting the cell cycle and inducing apoptosis in cancer cells.	Investigational anticancer context	β-CD, HP-β-CD, M-β-CD	Freeze drying	Initial burst followed by diffusion-controlled release	[91]
Bicalutamide	Inhibition of androgen receptor signaling.	Prostate cancer	HP-β-CD, SBE-β-CD	Freeze drying	Immediate release	[92]
Camptothecin	Inhibition of the enzyme topoisomerase I, which leads to DNA damage and cell death in cancer cells.	Anticancer drug development as a precursor of irinotecan and topotecan	Epi-β-CD, β-CD	Freeze drying	Rapid release; sustained release in peptide-grafted system	[93,94]
Clausenidin	Induction of apoptosis and inhibition of tumor growth.	Investigational anticancer context	HP-β-CD	Freeze drying	Pronounced sustained release	[95]
Curcumin	Inhibition of cancer cell proliferation via COX-2 and NF-κB pathway suppression.	Broad anticancer and chemopreventive research context	β-CD, HP-β-CD, γ-CD	Co-evaporation, co-precipitation, incubator shaking, kneading, solvent evaporation, freeze drying	Tunable release from rapid dissolution to pH- or enzyme-responsive release	[96,97,98,99,100,101]
Dasatinib	Targeting multiple kinases, inhibition of tumor growth and metastasis.	Leukemias and selected solid tumors	β-CD, HP-β-CD	Mechanochemical synthesis	Cyclodextrin-dependent release modulation	[102]
Diosmetin	Inhibition of cancer cell proliferation, induction of apoptosis, and suppression of metastasis through various cellular and molecular mechanisms.	Broad anticancer research context	β-CD	Co-grinding, kneading, microwave method	pH-dependent sustained release	[103]
Docetaxel	Stabilization of microtubules leading to mitotic arrest and apoptosis.	Breast, lung, prostate, gastric, and head and neck cancers	β-CD (+HPMC E5), alkylenediamine-modified β-CDs	Freeze drying, saturated aqueous solution	Biphasic sustained release; enzyme-responsive release	[104,105,106]
Doxorubicin	DNA intercalation, inhibition of topoisomerase II, production of ROS that cause DNA and cellular damage.	Breast cancer, leukemias, lymphomas, sarcomas, and other solid tumors	β-CD, D-glucose functionalized β-CD, D-maltose functionalized β-CD, β-CD-dendrimer	Freeze drying, co-precipitation	pH-responsive sustained release	[107,108,109,110,111]
Erlotinib	Locking the epidermal growth factor receptor’s tyrosine kinase activity, which signals cancer cells to multiply and grow.	Non-small cell lung cancer and pancreatic cancer	randomly methylated β-CD	Kneading, freeze drying	Immediate release	[112]
Etoposide	Inhibition of topoisomerase II, an enzyme essential for DNA replication and cell division.	Lung cancer, testicular cancer, lymphomas, and leukemias	β-CD	Kneading, solvent evaporation	Preparation method-dependent enhanced release	[113]
Exemestane	Irreversibly blocking the aromatase enzyme, significantly lowering estrogen levels, and slowing or stopping the growth of estrogen-dependent tumors.	Hormone receptor-positive breast cancer	β-CD (+HPMC E5)	Kneading, freeze drying	Enhanced release from ternary inclusion complexes	[114]
Formononetin	Inhibition of cancer cell growth, induction of programmed cell death, and suppression of metastasis across various cancers (breast, colon, prostate, etc.).	Broad anticancer research context	HP-β-CD	Neutralization agitation method	Sustained release after nanoparticle incorporation	[115]
Gefitinib	Selective inhibition of the tyrosine kinase activity of the epidermal growth factor receptor, which blocks the signaling pathways that cancer cells use for growth and survival.	EGFR-mutated non-small cell lung cancer	Epi-β-CD	Freeze drying	Rapid release	[116]
Lapatinib ditosylate	Blocking HER2 activity to suppress tumor cell proliferation.	HER2-positive breast cancer	β-CD (+PVP K-30)	Kneading, freeze drying	Enhanced release from ternary inclusion complexes	[117]
Methotrexate	Inhibition of dihydrofolate reductase, blocking DNA synthesis and cell proliferation, particularly in rapidly dividing cancer cells.	Leukemias, lymphomas, breast cancer, osteosarcoma, and other cancers	D-glucose functionalized β-CD, D-maltose functionalized β-CD, β-CD, HP-β-CD, M-β-CD, DM-β-CD, β-CD-(spacer-β-CD)_21_	Co-precipitation, spray drying	Rapid release; pH-responsive sustained release	[109,110,118,119]
Oridonin	Induction of apoptosis, inhibition of cell growth and invasion, promotion of autophagy, and overcoming of drug resistance.	Broad anticancer research context	HP-β-CD	Solvent evaporation	Immediate release	[120]
Paclitaxel	Stabilization of microtubules, disrupting their essential disassembly during cell division, which halts the cell cycle and leads to programmed cell death.	Breast, ovarian, lung, pancreatic, and Kaposi sarcoma	HP-β-CD, DM-β-CD	Solvent evaporation, freeze drying	Sustained release after nanoparticle incorporation	[121,122]
Pinostilbene	Inhibition of proliferation, induction of apoptosis, and prevention of metastasis.	Broad anticancer research context	α-CD, β-CD, γ-CD, M-β-CD, HP-β-CD	Kneading	pH-dependent enhanced release	[123]
Pomalidomide	Inducing apoptosis and cell cycle arrest in cancer cells, enhancing the immune system to fight cancer, and inhibiting blood vessel growth.	Multiple myeloma	SBE-β-CD	Freeze drying	Immediate release	[124]
Quercetin	Promoting apoptosis, inhibiting metastasis, regulating the cell cycle and inhibiting tumor angiogenesis.	Broad anticancer or chemopreventive research context	β-CD	Freeze drying	pH-responsive co-delivery with DOX	[107]
Raloxifene	Selective estrogen receptor modulator that blocks estrogen’s effects on cancer cells.	Breast cancer risk reduction and estrogen receptor-positive breast cancer context	β-CD, HP-β-CD	Physical mixing, co-precipitation	Limited but enhanced release	[125]
Resibufogenin	Induction of apoptosis, inhibition of cell cycle progression, and suppression of tumor angiogenesis in various cancer types.	Investigational anticancer context	β-CD, HP-β-CD	Solvent evaporation	Rapid and nearly complete release	[126]
Sorafenib	Blocking specific proteins, which slows the growth of blood vessels that tumors need to survive.	Hepatocellular, renal cell, and thyroid cancers	HP-β-CD	Co-precipitation	Rapid release	[127]
Tamoxifen	Blocking estrogen from fueling hormone receptor-positive breast cancer cells, halting their growth and triggering cell death.	Hormone receptor-positive breast cancer	M-β-CD, HP-β-CD, SBE-β-CD	Kneading, freeze drying	Rapid and complete release	[128]
Thymoquinone	Induction of apoptosis, inhibition of proliferation, and suppression of metastasis.	Broad anticancer research context	HP-β-CD	Freeze drying	Sustained release	[129]
Venetoclax	Inhibition of the BCL-2 protein, which promotes cancer cell survival and enhances the immune system’s ability to fight cancer.	Chronic lymphocytic leukemia, small lymphocytic lymphoma, and acute myeloid leukemia	HP-β-CD	Kneading	Sustained release	[130]

**Table 3:** Representative anticancer agents and MOF platforms for drug delivery (2015–2025).

Anticancer Agent	Anticancer Activity	Cancer Relevance	Carrier Type	Loading Strategy	Release Behavior	Ref.
5-fluorouracil	Interference with the metabolism of DNA and RNA, leading to cell death.	Colorectal, gastric, pancreatic, breast, and head and neck cancers	UCNP@ZIF-8/FA, Zn-BDC, 3D Nd(III)-MOF, CuMOP-N1, CuMOP-N2, Cu-MOF, Fe-MIL-53-NH_2_, MOC-19, MOC-22	Post-synthetic loading	pH-responsive release; sustained host–guest-mediated release	[157,158,159,160,161,162,163]
6-mercaptopurine	Inhibition of DNA/RNA synthesis and induction of tumor cell apoptosis.	Acute lymphoblastic leukemia and other hematologic malignancies	NMOF-5, ZIF-90@GOD@HA	Post-synthetic loading, one-pot encapsulation	pH-responsive sustained release	[164,165]
Aminopterin	Inhibition of dihydrofolate reductase, crucial for DNA synthesis, thereby arresting rapid cancer cell proliferation.	Antifolate-based anticancer research context	ZIF-90	Post-synthetic loading	pH-responsive prolonged release	[166]
Bortezomib	Induction of cancer cell death by disrupting protein degradation.	Multiple myeloma and mantle cell lymphoma	MMS@ZIF-8	Post-synthetic loading	pH-responsive sequential release	[167]
Bosutinib	Stopping leukemia cell growth, inducing apoptosis, effective against many imatinib-resistant CML forms.	Chronic myeloid leukemia	Ti-MOF/GO	Post-synthetic loading	pH-responsive controlled release	[168]
Carboplatin	Acting as an alkylating agent to induce cancer cell death by creating DNA adducts, which crosslink DNA strands to inhibit replication and transcription.	Ovarian, lung, head and neck, and other solid tumors	ZIF-8	Post-synthetic loading	pH-responsive controlled release	[169]
Camptothecin	Inhibition of the enzyme topoisomerase I, which leads to DNA damage and cell death in cancer cells.	Anticancer drug development as a precursor of irinotecan and topotecan	CoFe_2_O_4_@PDA@ ZIF-8, ZIF-8@RGD	Post-synthetic loading, one-pot encapsulation	pH-responsive targeted release; NIR-triggered release	[170,171]
Capecitabine	Acting as an antimetabolite to kill rapidly dividing cancer cells by converting into 5-FU, inhibiting DNA synthesis, and halting tumor growth.	Colorectal, breast, and gastric cancers	Sul-IRMOF-ACA-HA	Post-synthetic loading	pH-dependent sustained release	[172]
Cisplatin	Binding to DNA, forming platinum-purine adducts that distort the DNA structure, blocking replication and transcription.	Testicular, ovarian, bladder, lung, head and neck, and other solid tumors	UiO-66, UiO-66-NH_2_, UiO-66, UiO-67	Post-synthetic loading	MOF-drug interaction-dependent sustained release	[173,174]
Crizotinib	Inhibition of tumor cell proliferation, induction of apoptosis, and inhibition of angiogenesis.	ALK-positive and ROS1-positive non-small cell lung cancer	HA-ZIF-90@ICG	One-pot encapsulation	ATP-responsive release with PDT-assisted ROS generation	[175]
Curcumin	Inhibition of cancer cell proliferation via COX-2 and NF-κB pathway suppression.	Broad anticancer and chemopreventive research context	ZIF-8, MOF-Fe (DTBA) MOF-Al (DTBA), MOF-Zr (DTBA), glutamine-β-CD-MOF, M-lactose@ZIF-8-β-CD	One-pot encapsulation, post-synthetic loading	pH-responsive controlled release; GSH-responsive release	[176,177,178,179]
Cyclophosphamide	Interference with DNA replication and transcription by forming DNA crosslinks after metabolic activation in the liver.	Leukemias, lymphomas, breast cancer, ovarian cancer, and other cancers	MIL-100 (Fe)	Post-synthetic loading	Controlled release	[180]
Dacarbazine	Addition of alkyl groups to DNA, causing crosslinking that prevents division and leads to cell death.	Melanoma and Hodgkin lymphoma	MIL-100 (Fe)	One-pot encapsulation	PEG-modulated prolonged controlled release	[181]
Dasatinib	Targeting multiple kinases, inhibition of tumor growth and metastasis.	Leukemias and selected solid tumors	Series of Fe-, Zn-, and Zr-based MOFs	Post-synthetic loading	MOF structure-dependent release	[182]
Daunorubicin	Intercalation into DNA, inhibition of topoisomerase II, induction of apoptosis.	Acute leukemias	UiO-66-COOH- CAD-HA	Post-synthetic loading	pH-responsive targeted release	[183]
Docetaxel	Stabilization of microtubules leading to mitotic arrest and apoptosis.	Breast, lung, prostate, gastric, and head and neck cancers	nanoMIL-100 (Fe) UiO-66	Post-synthetic loading	pH-dependent sustained release prolonged by PEGylation	[184,185]
Doxorubicin	DNA intercalation, inhibition of topoisomerase II, production of ROS that cause DNA and cellular damage.	Breast cancer, leukemias, lymphomas, sarcomas, and other solid tumors	ZIF-7, ZIF-8, M-lactose@ZIF-8-β-CD, Zn-BTC, Fe-BTC, PPy@MIL-100, Fe_3_O_4_@Fe-MOF, PB@ZIF-8, UCNP@UiO-66-NH_2_/FA, Fe-MIL-88B-NH_2_, NMOF-DNA, CoFe_2_O_4_@PDA@ZIF-8, γ-CD-MOF	Post-synthetic loading, one-pot encapsulation	Multiple stimuli-responsive release, including pH-, ATP-, GSH-, and NIR-triggered systems	[170,179,186,187,188,189,190,191,192,193,194,195,196,197]
Epirubicin	Inhibition of tumor cell proliferation by intercalating DNA, inhibition of topoisomerase II, and induction of apoptosis.	Breast and gastric cancers	Fe_3_O_4_-Pt@MOF	Post-synthetic loading	pH-sensitive release with initial burst	[198]
Gemcitabine	Interference with DNA synthesis and repair, causing “masked termination” of DNA chains and inhibiting ribonucleotide reductase.	Pancreatic, lung, breast, ovarian, and bladder cancers	MIL-100 (Fe), ZIF-8, ZIF-67, ZIF-90, ZIF-92, ZIF-108	Post-synthetic loading	Phosphate- and ATP-triggered release	[199,200]
Imatinib	Induction of cancer cell growth arrest and apoptosis by targeting specific tyrosine kinases, primarily BCR-ABL, c-KIT, and PDGFR.	Chronic myeloid leukemia and gastrointestinal stromal tumors	MIL-100 (Fe), MIL-101 (Fe)-NH_2_	Post-synthetic loading	pH-dependent release with rapid acidic release	[201]
Lapatinib	Blocking HER2 activity to suppress tumor cell proliferation.	HER2-positive breast cancer	UiO-66	Post-synthetic loading	pH-responsive sustained release	[202]
Larotrectinib	Inhibition of tropomyosin receptor kinases encoded by *NTRK* gene fusions.	*NTRK* fusion-positive solid tumors	Fe-MOF	Post-synthetic loading	Extended release	[203]
Letrozole	Suppression of estrogen synthesis by blocking the conversion of androgens to estrogens, thereby inhibiting the growth of estrogen-dependent breast cancer.	Hormone receptor-positive breast cancer	ZIF-8@CS-FA, UiO-66, UiO-66@NH_2_	One-pot encapsulation, post-synthetic loading	pH-dependent gradual controlled release	[204,205]
Lonidamine	Inhibition of energy metabolism in tumor cells, primarily through inhibiting hexokinase-2 and mitochondrial respiration.	Investigational anticancer and tumor metabolism research context	ZIF-8, ZIF-67, ZIF-90, ZIF-92, ZIF-108	Post-synthetic loading	ATP-triggered sustained release	[200]
Methotrexate	Inhibition of dihydrofolate reductase, blocking DNA synthesis and cell proliferation, particularly in rapidly dividing cancer cells.	Leukemias, lymphomas, breast cancer, osteosarcoma, and other cancers	ZJU-64, ZJU-64-CH_3_, Zn-TBDA, Zn-GA	Post-synthetic loading, one-pot encapsulation	pH- and temperature-responsive sustained release	[206,207,208]
Norcantharidin	Inhibition of cell proliferation, induction of apoptosis and autophagy, and prevention of metastasis.	Investigational anticancer context	IRMOF-3	Post-synthetic loading	Thermosensitive gel-mediated sustained release	[209]
Oridonin	Triggering apoptosis, stopping cell growth, promoting autophagy, inhibiting invasion, and overcoming drug resistance.	Broad anticancer research context	PCN-222, MIL-53 (Fe), MOF-5	Post-synthetic loading	Sustained release with formulation-dependent pH sensitivity	[210,211,212]
Oxaliplatin	Its platinum core forms DNA adducts, primarily intra-strand crosslinks, which block DNA replication and transcription, leading to cancer cell death.	Colorectal cancer and gastrointestinal cancers	UiO-66-NH_2_, UiO-66-NH_2_-FA	Post-synthetic loading	Folate-targeted sustained release with reduced burst effect	[213]
Paclitaxel	Stabilization of microtubules, disrupting their essential disassembly during cell division, which halts the cell cycle and leads to programmed cell death.	Breast, ovarian, lung, pancreatic, and Kaposi sarcoma	UiO-66, UiO-67, Fe_3_O_4_@ IRMOF-3/FA, MIL-53 (Fe)	Post-synthetic loading	Sustained release; pH- and magnetothermal-responsive release	[174,214,215]
Piperlongumine	Induction of cancer cell death, inhibition of cell proliferation, and inhibiting metastasis.	Broad anticancer research context	Tf-LipoMOF	Post-synthetic loading	pH-responsive release with improved retention at physiological pH	[216]
Quercetin	Promotion of apoptosis, regulation of the cell cycle, inhibition of metastasis and tumor angiogenesis.	Broad anticancer and chemopreventive research context	UiO-66, UiO-66-NH_2_, UiO-66-NO_2_	Post-synthetic loading	pH-responsive prolonged release	[217]
Sorafenib	Inhibition of tumor angiogenesis by blocking proteins required for blood vessel growth.	Hepatocellular, renal cell, and thyroid cancers	MX-UiO-67, MIL-53 (Fe)	Post-synthetic loading	pH- and NIR-responsive release; sustained release	[218,219]
Tamoxifen citrate	Inhibition of tumor growth by binding to estrogen receptors, blocking estrogen’s proliferative signals, and inducing apoptosis.	Hormone receptor-positive breast cancer	AgNPs@MOF-808, MOF-808	Post-synthetic loading	Sustained release with initial burst	[220]
Temozolomide	Damaging cancer cell DNA, primarily through methylating guanine, leading to cell death (standard treatment for brain tumors).	Glioblastoma and other malignant gliomas	MIL-53 (Fe), ZIF-8, TA@ZIF-8	Post-synthetic loading, one-pot encapsulation	pH- and hyperthermia-responsive controlled release	[215,221]
Tirapazamine	Activation at the low oxygen levels found in solid tumors, killing poorly oxygenated or hypoxic cells.	Hypoxia-targeted anticancer research context	MSN@carMOF	One-pot encapsulation	Glucose oxidase-mediated pH-triggered and hypoxia-activated release	[222]
Topotecan	Interference with DNA replication by stabilizing the topoisomerase I-DNA complex, leading to DNA strand breaks and cell death.	Ovarian, cervical, and small cell lung cancers	CL-MOF	Post-synthetic loading	Sustained release with reduced burst and lactone stabilization	[223]
Triptolide	Inhibition of tumor cell proliferation, induction of apoptosis, suppression of invasion and metastasis, and modulation of inflammatory and cancer-related signaling pathways.	Hepatocellular carcinoma and broad anticancer research context	γ-CD-MOF	Post-synthetic loading	Slower release with enhanced solubility	[224]

**Table 4:** Comparative overview of key features, limitations, release behavior, stability, and translational applicability of the three formulation platforms discussed in this review [133,239,240,241,242].

Comparative Aspect	Polymeric Solid Dispersions	Cyclodextrin-Based Inclusion Complexes	Metal–Organic Frameworks
**Key** **Advantages**	Improved dissolution rate and bioavailability through reduced particle size, improved wettability, increased porosity, and stabilization of the drug in an amorphous state.	Improved solubility, drug stability, controlled release, and cellular uptake through inclusion complex formation, with potential for stimuli-responsive and targeted delivery.	Highly tunable porous structures with high loading capacity, enabling controlled, stimuli-responsive, multifunctional, and theranostic delivery.
**Main** **Limitations**	Physical instability due to possible recrystallization of the amorphous drug, moisture sensitivity, phase separation, limited reproducibility, and challenges in scale-up and incorporation into final dosage forms.	Limited by cavity size, binding affinity, complex stoichiometry, and possible dose-related safety concerns for some derivatives. Release behavior can be difficult to generalize.	Limited clinical translation due to toxicity concerns, biological stability, degradability, long-term biosafety, reproducibility, scalable synthesis, and regulatory complexity.
**Release** **Characteristics**	Enhanced or immediate release through amorphization and supersaturation, or controlled release by dissolution-, diffusion-, or erosion-controlled mechanisms depending on carrier properties.	Immediate, rapid, sustained, or stimuli-responsive release depending on cyclodextrin type, derivative, preparation method, and formulation architecture.	Broad release modulation, including sustained, controlled, pH-, GSH-, ATP-, enzyme-, NIR-, temperature-, and hypoxia-responsive release.
**Stability** **Considerations**	Risk of recrystallization, phase separation, and moisture-induced instability of amorphous systems.	Improved drug stability through protection from environmental degradation, although the effect depends on the drug, cyclodextrin type, and complex composition.	Stability depends on metal-ligand coordination, framework composition, particle size, surface chemistry, and biological environment.
**Translational** **Applicability**	Relatively high translational readiness due to established pharmaceutical use and compatibility with conventional processing, although drug-specific optimization and scale-up may remain challenging.	Good translational potential due to established pharmaceutical use and broad therapeutic applicability, although the number of commercially available products remains limited and further safety, scale-up, and regulatory optimization is needed.	Mostly preclinical, although selected MOF-based systems have entered clinical trials as radiosensitizers. Further translation requires validated safety, degradation and clearance profiles, reproducible production, and scalable green synthesis.

## Data Availability

No new data were created or analyzed in this study. Data sharing is not applicable to this article.

## Abbreviations

The following abbreviations are used in this manuscript:

5-FU	5-fluorouracil
α-CD	α-cyclodextrin
β-CD	β-cyclodextrin
γ-CD	γ-cyclodextrin
BCS	Biopharmaceutical Classification System
DM-β-CD	Dimethyl-β-cyclodextrin
DOX	Doxorubicin
Epi-β-CD	Epichlorohydrin-crosslinked β-cyclodextrin
FDA	U.S. Food and Drug Administration
GRAS	Generally Recognized as Safe
GSH	Glutathione
HP-β-CD	Hydroxypropyl-β-cyclodextrin
HPMC	Hydroxypropyl methylcellulose
L-SEDDS	Liquid self-emulsifying drug delivery systems
M-β-CD	Methyl-β-cyclodextrin
MOF	Metal–organic framework
MPEG-PCL	Polyethylene glycol-poly(ε-caprolactone) micelles
MTX	Methotrexate
Na-CMC	Sodium carboxymethylcellulose
NIR	Near-infrared light
PEG	Polyethylene glycol
PVP	Polyvinylpyrrolidone
ROS	Reactive oxygen species
SBE-β-CD	Sulfobutylether-β-cyclodextrin
SDS	Sodium dodecyl sulfate
S-SEDDS	Solid self-emulsifying drug delivery systems
TEM	Transmission electron microscopy
TPGS	D-α-tocopheryl polyethylene glycol succinate
UCNP	Upconversion nanoparticle
